# Combination of gene/protein and metabolite multiomics to reveal biomarkers of nickel ion cytotoxicity and the underlying mechanism

**DOI:** 10.1093/rb/rbae079

**Published:** 2024-06-29

**Authors:** Yan Huang, Fudan Zhang, Yajing Zhang, Rong Chen, Xiaoying Lü

**Affiliations:** State Key Laboratory of Digital Medical Engineering, School of Biological Science and Medical Engineering, Southeast University, Nanjing 210096, China; State Key Laboratory of Digital Medical Engineering, School of Biological Science and Medical Engineering, Southeast University, Nanjing 210096, China; State Key Laboratory of Digital Medical Engineering, School of Biological Science and Medical Engineering, Southeast University, Nanjing 210096, China; State Key Laboratory of Digital Medical Engineering, School of Biological Science and Medical Engineering, Southeast University, Nanjing 210096, China; State Key Laboratory of Digital Medical Engineering, School of Biological Science and Medical Engineering, Southeast University, Nanjing 210096, China

**Keywords:** cytotoxicity of nickel ion (Ni^2+^), multiomics integrative analysis, gene/protein/metabolite biomarkers, molecular mechanisms

## Abstract

Biomarkers have been applied for toxicity assessment of biomaterials due to their advantages. However, research on biomarkers for biomaterials is still in its early stages. There is a lack of integrated analysis in biomarker research based on multiomics studies. Herein, we report a new approach for combining of gene/protein and metabolite multiomics to reveal biomarkers of nickel ion (Ni^2+^) cytotoxicity and the underlying mechanism. Firstly, differentially expressed genes and proteins were compared to screen gene/protein pairs exhibiting consistent differential expression within the same Ni^2+^-treated groups. Next, metabolic pathway analysis was carried out to reveal pathways in which gene/protein pairs and metabolites showed upstream and downstream relationships. Important networks composed of gene/protein pairs, metabolites and metabolic pathways and candidate biomarkers were subsequently identified. Through expression level and function validation, the gene/protein/metabolite biomarkers were confirmed, and the underlying mechanism was revealed: Ni^2+^ influenced the expression of the *Rrm2* gene biomarker, which subsequently affected the expression of the RRM2 protein biomarker. These changes in turn impacted the levels of uric acid and uridine metabolite biomarkers, ultimately inhibiting DNA synthesis, suppressing cell proliferation, increasing intracellular ROS levels and reducing ATP content.

## Graphical Abstract

**Figure:**
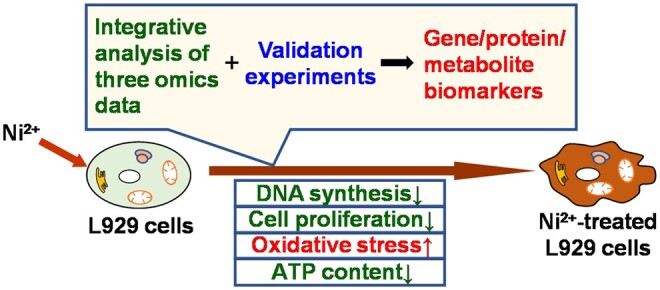

## Introduction

Biomarkers have been applied for toxicity assessment of biomaterials due to their advantages, such as rapid detection, simplicity and short time requirements [1–3]. However, research on biomarkers for biomaterials is still in its early stages. Several researchers have employed traditional molecular biology methods and conducted experiments by applying known cancer or toxicity biomarkers from other fields into the field of biomaterials [4, 5]. Few researchers have utilized transcriptomic and proteomic methods to study gene/protein biomarkers of biomaterials [6–8].

The interaction between biomaterials and cells is complex and involves changes in multiple genes and proteins [9–12]; these interactions impact many cellular activities at the metabolic level. Processes such as cell signalling release, energy transfer and intercellular communication are all regulated by metabolites [13, 14]. Therefore, both intermediate and end products of abnormal metabolic processes may serve as biomarkers to indicate the safety of biomaterials. However, due to the inability to predict changes in metabolites through changes in gene/protein expression and the complex regulatory interactions between metabolites and genes/proteins, further research on metabolite biomarkers is needed to provide a basis for gene/protein biomarker studies. The emergence of metabolomics technologies has accelerated the study of metabolite biomarkers. One study [15] utilized metabolomics to screen for serum biomarkers in workers exposed to TiO_2_ nanoparticles, and 296 differentially expressed serum metabolites were revealed. Furthermore, eight potential metabolite biomarkers were identified through three machine learning methods (random forest, support vector machine and Boruta).

There is a tight upstream–downstream relationship among genes, proteins and metabolites. The impact of biomaterials on cellular behaviour is inevitably a highly complex process that involves multiple molecules (genes/proteins/metabolites), manifests at multiple levels (transcriptional/protein/metabolic level), and exerts effects through various biological pathways. However, current studies on toxicological biomarkers of biomaterials are limited predominantly to the initial screening stage using a single omics approach [6–8, 15]. There is a lack of integrated analysis in biomarker research for biomaterials based on multiomics studies, leading to inconsistencies in biomarkers across the gene/protein/metabolite levels for the same material. On the other hand, recent studies on biomarkers of biomaterials are limited to the theoretical screening stage. There has been no further molecular biology based experimental validation of the potential biomarkers. Therefore, these screened genes/proteins/metabolites cannot yet be confirmed as cytotoxicity biomarkers of biomaterials.

While nickel-titanium alloys are widely used in clinical treatment, numerous studies have indicated that the release of Ni^2+^ after implantation can lead to allergic reactions and toxic effects. Therefore, the safety of Ni^2+^ and its interaction mechanisms with cells have been a subject of high interest among researchers [16, 17]. Some researchers have employed traditional molecular biology methods to study the cytotoxicity biomarkers of Ni^2+^ [18, 19], but as of now, there have only been reports on the use of gene chip/transcriptome sequencing and proteomics for screening [1, 20–22].

In a study mentioned in reference [22], gene chips and functional proteomics were used to investigate the gene and protein expression profiles of human colon cancer cells (RKO) treated with a low dose of toxic metal (20 μM Ni^2+^) for 24 h. Subsequently, pathway analysis identified 26 genes and 16 proteins involved in toxicity-related pathways, such as cell apoptosis, differentiation, proliferation and tumorigenesis. Researchers have suggested that these genes and proteins could serve as biomarkers for assessing the cytotoxicity of Ni^2+^. However, the results of these two omics experiments were analysed separately and without comprehensive analysis, leading to discrepancies in the expression of 12 biomarkers at the gene/protein levels. Furthermore, since further molecular biology experimental validation of the screened potential biomarkers was not conducted, whether these 26 genes and 16 proteins were biomarkers has not been confirmed. Over the years, our research group has employed cDNA microarray/transcriptome sequencing, proteomics and metabolomics technologies to investigate the toxic effects of Ni^2+^ on L929 cells [23–25]. We have published the only paper to date that utilizes the integrated screening of transcriptomics/proteomics data and validates the expression levels and function of target biomarkers to identify cytotoxicity biomarkers of Ni^2+^ [1].

However, although integrative transcriptomics and proteomic studies could ameliorate the inconsistency of biomarkers at the genetic/protein level, due to the lack of metabolomics data, constructing a regulatory network consisting of intracellular gene/protein pairs, metabolites and metabolic pathways has not been possible. Hence, the regulatory effects of upstream gene/protein biomarkers on downstream metabolite biomarkers have not been elucidated.

Therefore, the purpose of this article is to continue this research based on our preliminary studies, which focused on Ni^2+^, and conduct an integrative investigation of gene/protein/metabolite biomarkers. First, metabolomics studies on the interaction between Ni^2+^ and L929 cells were carried out. Then, an integrative analysis of transcriptomic, proteomic and metabolomic data was performed to identify for gene/protein pairs and metabolites that were differentially expressed in the same Ni^2+^-treated group, participated in the same metabolic pathways and demonstrated upstream–downstream relationships. Therefore, important gene/protein pairs, metabolites and metabolic pathway networks were constructed, and candidate gene/protein/metabolite biomarkers were identified. Next, stable cell lines with target gene silencing/overexpression were constructed through RNA interference (RNAi) and gene overexpression techniques. The changes in candidate gene/protein/metabolite biomarkers were validated through qRT–PCR, western blotting and liquid chromatography–mass spectrometry (LC–MS/MS); the correlated target gene/protein/metabolite biomarkers of Ni^2+^-induced cytotoxicity were determined, and a regulatory network of gene transcription, protein expression and metabolite alteration was established. Subsequently, functional validation of the above target gene/protein/metabolite biomarkers was carried out, and the genes/proteins/metabolites biomarkers associated with Ni^2+^-induced cytotoxicity were confirmed through cell cycle analysis, cell proliferation assays, reactive oxygen species (ROS) analysis and ATP content determination. Finally, the mechanisms of action of these biomarkers in Ni^2+^-induced cytotoxicity were explored.

## Materials and methods

### Preparation of materials

Nickel chloride hexahydrate powder (Sinopharm Chemical Reagent Co., Ltd) was dissolved in RPMI-1640 complete medium (89% RPMI-1640 from Gibco, USA, 10% foetal bovine serum from Hangzhou Sijiqing Biological Products Co., Ltd, 1% penicillin–streptomycin from Gibco) to achieve final concentrations of 100 and 200 μM of Ni^2+^.

### Cultivation of L929 cells

L929 mouse fibroblast cells were obtained from Shanghai Cell Bank of the Chinese Academy of Sciences. The cells were cultured in RPMI-1640 complete medium at 37°C in a humidified atmosphere of 5% CO_2_. Cells in the logarithmic growth phase were selected for subsequent experiments.

### Biomics experiments

#### Transcriptomic and proteomic experiments

In previous studies, our research group utilized transcriptomic sequencing and iTRAQ-based proteomic techniques to investigate the gene and protein expression profiles of L929 cells treated with 100 and 200 μM Ni^2+^ for 12, 24 and 48 h. Differentially expressed genes and proteins were identified in each experimental group [1, 25]. In this article, we further selected gene/protein pairs that exhibited consistent differential expression (upregulated or downregulated) within the same Ni^2+^-treated group.

#### Metabolomic experiment

To perform multiomics integrative analysis with the previously obtained transcriptomics and proteomics results, the experimental conditions for metabolomics were the same as those used in the previous experiments. L929 cells were seeded in cell culture flasks with a bottom area of 75 cm^2^. The number of seeded cells was 4 × 10^6^, 3 × 10^6^ or 1.5 × 10^6^ in the experimental groups (cells treated with 100 or 200 μM Ni^2+^ for 12, 24 or 48 h, respectively) and 10^6^ in the control group (cells not treated with Ni^2+^ and cultured for 72 h).

Metabolomic analysis was performed using LC-Q/TOF-MS-based metabolomics techniques [13, 14, 25]. The impact of 100 and 200 μM Ni^2+^ on the metabolite profile of L929 cells after treatment for 12, 24 and 48 h was analysed, and differentially abundant metabolites were selected by comparison to those in the control group.

### Screening of candidate gene/protein/metabolite biomarkers

The consistent differentially expressed gene/protein pairs obtained in ‘Transcriptomic and proteomic experiments’ section and the differential metabolites obtained in ‘Metabolomic experiment’ section were subjected to pathway analysis using MetaboAnalyst (http://www.metaboanalyst.ca). The analysis was performed separately for each Ni^2+^-treated group, and pathways involving gene/protein pairs and metabolites were identified. Furthermore, pathways exhibiting associated with gene/protein pairs and metabolites exhibiting upstream–downstream relationships were selected. A network composed of gene/protein pairs and metabolites and metabolic pathways was constructed using Cytoscape 3.7.2, and candidate gene/protein/metabolite biomarkers were subsequently screened.

### Validation of candidate gene/protein/metabolite biomarkers

#### Construction of stable cell lines for gene silencing/overexpression

RNAi and gene overexpression techniques were employed to generate stable *Rrm2-*silenced/*Rrm2-*overexpressing L929 cell lines. The expression of the target *Rrm2* gene in the constructed cell lines was validated through qRT–PCR. The empty vector-transfected L929 cells served as the *Rrm2*-silenced L929 cell control, and the empty virus-transfected L929 cells served as the *Rrm2*-overexpressing L929 cell control. The above experiments were conducted by Shanghai Omicsspace Biotech Co., Ltd.

#### Validation of the expression levels of candidate genes/proteins/metabolites biomarkers

##### qRT-PCR analysis

Four types of cells (*Rrm2-*silenced control cells, *Rrm2-*silenced L929 cells, *Rrm2*-overexpressing control cells and *Rrm2*-overexpressing L929 cells) were seeded in 25 cm^2^ cell culture flasks. The cells were treated with 100 or 200 μM Ni^2+^ for 12 h. Total RNA was extracted from the treated or untreated cells (cultured in a complete medium without Ni^2+^), and qRT-PCR was performed to measure the expression levels of *Rrm2* in each group. GAPDH was used as an internal reference.

##### Western blotting analysis

Total protein was extracted from the Ni^2+^-treated and untreated cells (cell culture and treatment were the same as those described in the ‘qRT-PCR analysis’ section). Western blotting analysis was performed to measure the expression levels of RRM2 in each group. GAPDH was used as an internal reference.

##### LC-MS/GC-MS detection

Total intracellular metabolites were extracted from Ni^2+^-treated and untreated cells (cell culture and treatment were the same as those described in the ‘qRT-PCR analysis’ section). LC-MS/MS (AB SCIEX ExionLC chromatography and AB SCIEX Q-TRAP 5500+ mass spectrometer) was used to quantify the levels of four metabolites (uric acid, deoxyguanosine 5′-monophosphate (dGMP), deoxyuridine monophosphate (dUMP) and uridine) in each group.

#### Functional validation of target gene/protein/metabolite biomarkers

##### Cell cycle analysis

Four cell types (the same as those described in the ‘qRT-PCR analysis’ section) were seeded in 25 cm^2^ cell culture flasks. After 12 h of treatment with 100 or 200 μM Ni^2+^, the cells were digested with trypsin, collected, fixed with ice-cold ethanol, stained with propidium iodide and analysed for cell cycle distribution using flow cytometry (FACSCalibur, BD, USA) [26]. ModFitLT 3.2 software was used to determine the percentage of cells in each phase (G1, S and G2/M). Cells cultured in complete medium without Ni^2+^ served as the untreated control for each cell type.

##### Cell proliferation rate determination

L929 cell suspensions for each type of cell (the cell types used were the same as those described in the ‘qRT–PCR analysis’ section) were added to 96-well plates (6 × 10^4^ cells/ml, 100 μl/cell). After 48 h, the culture medium was removed, 200 μl of 100 or 200 µM Ni^2+^ was added, and the cells were further cultured for 12 h. An MTT assay was performed to measure the effects of Ni^2+^ on the proliferation of these four types of L929 cells [23]. The conditions for the untreated groups were the same as those described in the ‘Cell cycle analysis’ section.

##### Oxidative stress analysis

The cell type, cell culture and treatment process, as well as the setup of the Ni^2+^ treatment and untreated groups, were the same as those described in the ‘Cell proliferation rate determination’ section. After culturing for 12 h, the intracellular ROS production in each sample was measured using a ROS assay kit (Beyotime, China) according to the manufacturer’s protocols [27]. The fluorescence intensity of 2′,7′-dichlorofluorescein (DCF) was measured using a high-content cell analysis system (ArrayScan XTI, Thermo Scientific, USA) with an excitation wavelength of 485 nm and an emission wavelength of 528 nm.

##### Measurement of ATP content

The ATP content in cells was determined using an ATP assay kit (Beyotime, China). L929 cell suspensions for each type of cell (the cell types used were the same as those described in the ‘qRT–PCR analysis’ section) were added to 24-well plates (6 × 10^4^ cells/ml, 1 ml/cell). After 48 h, the culture medium was replaced with 1.5 ml of 100 or 200 µM Ni^2+^. After 12 h of treatment, the cells were lysed, and the supernatant was collected. The ATP concentration (*C*_ATP_) and protein concentration (*C*_protein_) of each sample were determined, and the ratio of ATP concentration to protein concentration (*C*_ATP_/*C*_protein_) was calculated [28]. The setup of the untreated groups was the same as that described in the ‘Cell cycle analysis’ section.

### Statistical analysis

The experimental data are presented as the mean±standard deviation (mean±SD). Unless otherwise stated, statistical analysis was performed using Student’s *t* test. *P* < 0.05 was considered statistically significant, and *P* < 0.01 was considered highly significant. The experiments were performed at least three times.

## Results and discussion

As L929 cells are the preferred cells in the standard of ‘Biological evaluation of medical devices-Part 5: Tests for *in vitro* cytotoxicity’ (ISO 10993-5, GB/T16886-5), so they were chosen as the experimental cell line in this paper.

### Biomics experiment results

#### Transcriptomics and proteomics experiment results

The differentially expressed genes and proteins in L929 cells after treatment with 100 or 200 μM Ni^2+^ for 12, 24 or 48 h were compared within each experimental group, and gene/protein pairs showing consistent expression patterns in the same group were identified. There were 12, 9 and 22 gene/protein pairs in the 12, 24 and 48 h groups treated with 100 μM Ni^2+^, respectively. In the 12, 24 and 48 h groups treated with 200 μM Ni^2+^, there were 13, 7 and 17 gene/protein pairs, respectively. The detailed information is listed in Supplementary Table S1.

#### Metabolomics experiment results

The number of differentially abundant metabolites in L929 cells after treatment with 100 or 200 μM Ni^2+^ for 12, 24 or 48 h is shown in Table 1. The detailed information is listed in Supplementary Tables S2–S7 and includes a total of 218 metabolites.

**Table 1.** The number of differential metabolites in L929 cells after being treated with Ni^2+^

Group	The differential metabolites with content increased	The differential metabolites with content decreased	Total differential metabolites
100 μM Ni^2+^-12 h	27	24	51
100 μM Ni^2+^-24 h	20	28	48
100 μM Ni^2+^-48 h	16	11	27
200 μM Ni^2+^-12 h	17	31	48
200 μM Ni^2+^-24 h	34	32	66
200 μM Ni^2+^-48 h	49	11	60

### Screening of candidate gene/protein/metabolite biomarkers

Metabolic pathway analysis integrates metabolomic data analysis results with biological knowledge. This approach integrates information on metabolite changes into biologically meaningful metabolic pathways. By analysing pathways involving specific genes, proteins and metabolites, identifying the most important differential metabolic pathways from extensive metabolic pathway databases is possible. The numbers of metabolic pathways associated with the gene/protein pairs and metabolites in the Ni^2+^-treated groups are shown in Table 2.

**Table 2.** The number of metabolic pathways in which gene/protein pairs and metabolites involved in the interaction between L929 cells and Ni^2+^

Group	Pathway that gene/protein pairs and metabolites involved	Pathway that gene/protein pairs and metabolites showing upstream-downstream relationships
100 μM Ni^2+^-12 h	4	2
100 μM Ni^2+^-24 h	2	1
100 μM Ni^2+^-48 h	0	0
200 μM Ni^2+^-12 h	4	4
200 μM Ni^2+^-24 h	2	0
200 μM Ni^2+^-48 h	2	2

In the regulation of biological systems, metabolites are situated downstream of gene regulation, and subtle variations in gene and protein expression can be amplified via metabolite expression; therefore, metabolomics results can serve as a validation of upstream omics regulatory outcomes. Based on this premise, pathways in which gene/protein pairs and metabolites exhibited upstream–downstream relationships were further analysed, and the results are presented in Table 2, with detailed information available in Table 3.

**Table 3.** The detailed information on the pathways with gene/protein pairs and metabolites showing upstream–downstream relationships in the interaction between Ni^2+^ and L929 cells

Group	Metabolic pathway	Gene/protein pair	Metabolite
100 μM Ni^2+^-12 h	① Purine metabolism pathway	*Rrm2*/RRM2 **↑**	Uric acid ↑
	② Biosynthesis of unsaturated fatty acids pathway	*Acot2*/ACOT2 **↓**	Palmitic acid ↑ Oleic acid ↑ Linoleic acid ↓
100 μM Ni^2+^-24 h	① Cysteine and methionine metabolism pathway	*Mat2a*/MAT2A **↓**	3-Sulfinylpyruvate ↓
200 μM Ni^2+^-12 h	① Purine metabolism pathway	*Rrm2*/RRM2 **↑**	dGMP ↓
	② Pyrimidine metabolism pathway	*Rrm2*/RRM2 **↑**	Uridine ↓ dUMP ↑
	③ Amino sugar and nucleotide sugar metabolism pathway	*Gnpnat1*/GNPNAT1 **↓**	D-Glucose 1-phosphate ↑
	④ Porphyrin metabolism pathway	*Cp*/CP **↓**	Bilirubin ↓
200 μM Ni^2+^-48 h	① Cysteine and methionine metabolism pathway	*Mat2a*/MAT2A **↓**	5'-Methylthioadenosine ↑ L-Cystathionine ↑
	② Glycerophospholipid metabolism pathway	*Gpd1*/GPD1**↓**	sn-Glycero-3-phosphocholine ↑

Our previous research showed that the cell proliferation of L929 cells treated with Ni^2+^ were 96.9% (100 μM Ni^2+^-12 h), 92.7% (100 μM Ni^2+^-24 h), 90.1% (100 μM Ni^2+^-48 h), 96.1% (200 μM Ni^2+^-12 h), 89.3% (200 μM Ni^2+^-24 h) and 81.8% (200 μM Ni^2+^-48 h) [1]. The Pearson correlation coefficients (*R*) were then calculated between cell proliferation rate and the number of differential metabolites (Table 1), metabolic pathways that gene/protein pairs and metabolites jointly involved, metabolic pathway that gene/protein pairs and metabolites showing upstream-downstream relationships (Tables 2 and 3). The results showed that in the 100 μM Ni^2+^ treatment group, there was a strong correlation (*R* > 0.8) between cell proliferation rate and all three indicators mentioned above. However, in the 200 μM Ni^2+^ treatment group, cell proliferation rate was only highly correlated with the number of metabolic pathways that gene/protein pairs and metabolites jointly involved.

**Figure 1.**
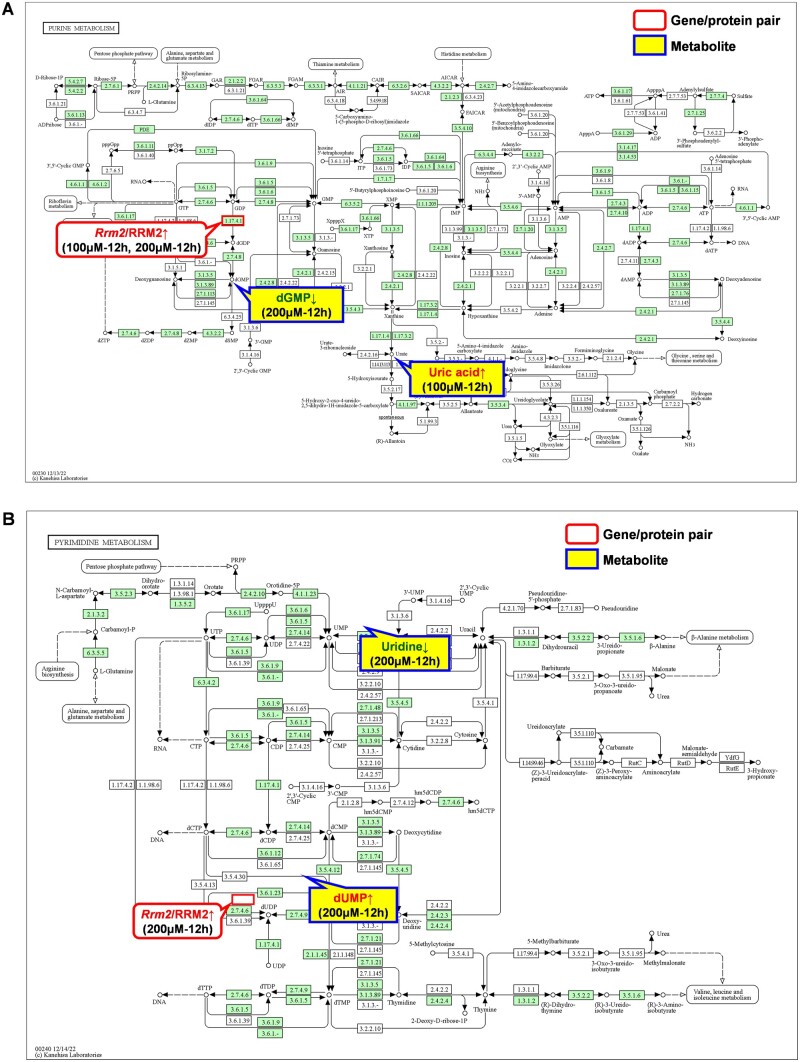
The purine metabolism pathway (**A**) and the pyrimidine metabolism pathway (**B**) [29] in which gene/protein pair and metabolites exhibited upstream-downstream relationships in the interaction between Ni^2+^ and L929 cells.

According to Table 3, there were a total of seven pathways in which the gene/protein pairs and metabolites exhibited upstream–downstream relationships. These pathways included (i) the purine metabolism pathway (Figure 1A), (ii) the biosynthesis of unsaturated fatty acids pathway (Supplementary Figure S1), (iii) the cysteine and methionine metabolism pathway (Supplementary Figure S2), (iv) the pyrimidine metabolism pathway (Figure 1B), (v) the amino sugar and nucleotide sugar metabolism pathway (Supplementary Figure S3), (vi) the porphyrin metabolism pathway (Supplementary Figure S4), and (vii) the glycerophospholipid metabolism pathway (Supplementary Figure S5). These pathways included a total of 6 gene/protein pairs and 13 metabolites.

**Figure 2.**
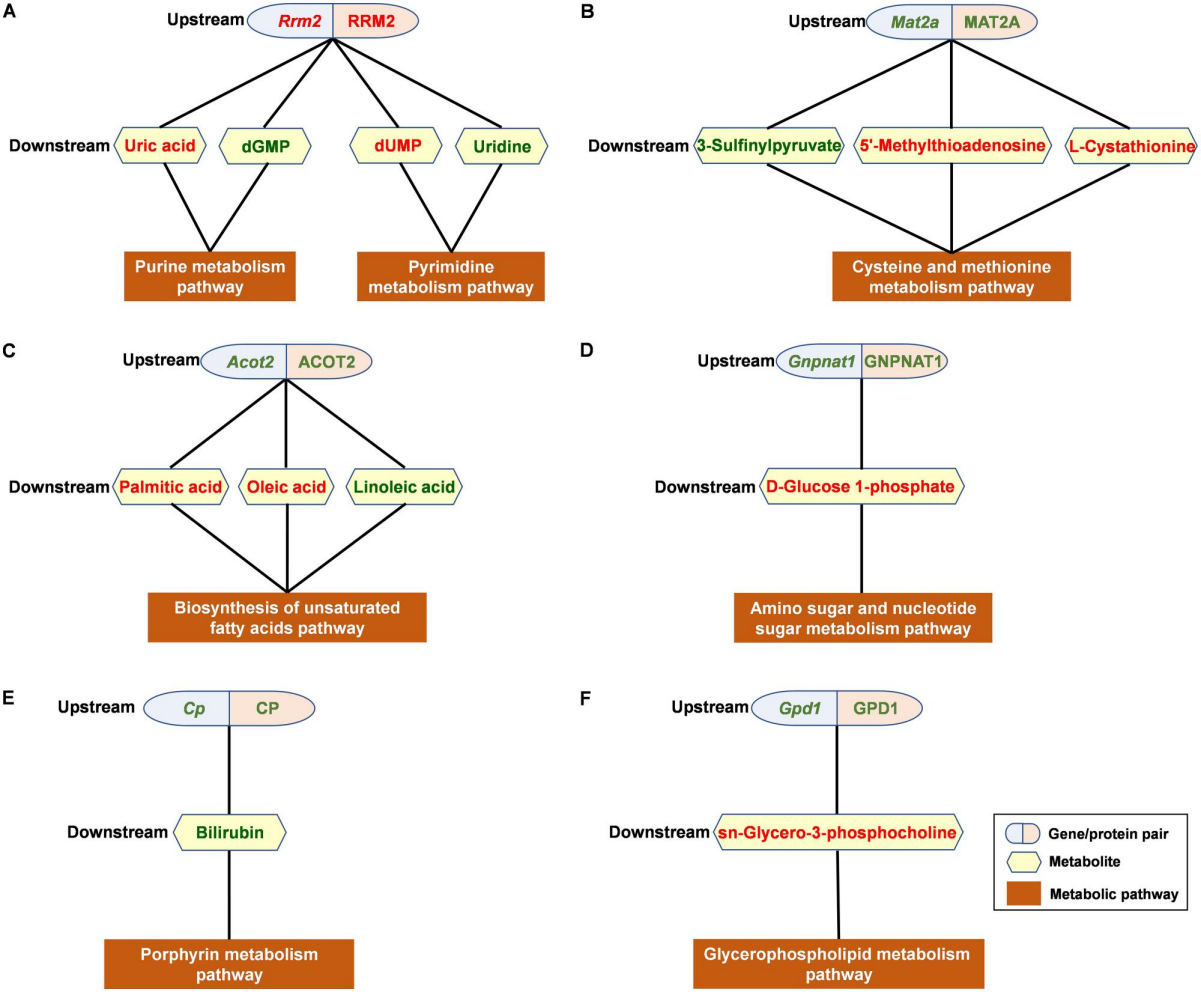
The network composed of gene/protein pairs and metabolites and metabolic pathways in the interaction between Ni^2+^ and L929 cells. (**A**)–(**F**) were six subnetworks contained.

The interactions among genes, proteins and metabolites within cells can be used to form a regulatory network. The construction of a network composed of gene/protein pairs and metabolite and metabolic pathways enable the determination of interactions among these components and study of the regulatory mechanisms of upstream gene/protein pairs on downstream metabolites. Therefore, based on the results shown in Table 3, a gene/protein pair, metabolite and metabolic pathway network consisting of 6 subnetworks was constructed using Cytoscape (Figure 2; Table 4). Among them, subnetwork A encompassed the highest number of metabolites and metabolic pathways; therefore, in this article, we focused on subnetwork A.

**Table 4.** The subnetworks contained in the network are composed of gene/protein pairs and metabolites and metabolic pathways in the interaction between Ni^2+^ and L929 cells

Subnetwork	Number of gene/protein pair	Number of metabolites	Number of metabolic pathways
A	1	4	2
B	1	3	1
C	1	3	1
D	1	1	1
E	1	1	1
F	1	1	1

Subnetwork A (Figure 2A) involved two metabolic pathways: the purine metabolism pathway (Figure 1A) and the pyrimidine metabolism pathway (Figure 1B). Purine metabolism refers to the biosynthesis and decomposition of purine derivatives such as adenine and guanine. Several enzymes participate in the synthesis and decomposition of purines, and abnormalities in these enzymes can disrupt metabolism, leading to increased uric acid synthesis or reduced excretion. An increase in certain purine metabolites (such as xanthine and urea) may indicate mitochondrial stress [30]. Pyrimidine metabolism refers to a series of metabolic pathways in which pyrimidines (or their derivatives) are used as substrates or metabolic products. This process is also known as *de novo* synthesis of pyrimidine nucleotides and is crucial for the biosynthesis of pyrimidine nucleotides. In this pathway, orotic acid is synthesized first, followed by glycosidic bond formation with ribose-5-phosphate to form orotidine monophosphate (OMP). OMP can be further modified to generate the first pyrimidine nucleotide product, uridine monophosphate (UMP). UMP can undergo further phosphorylation and uracil modification to ultimately be converted into cytidine triphosphate (CTP). These two metabolic pathways include one upstream gene/protein pair (Rrm2/RRM2) that regulated four downstream metabolites (uric acid, dGMP, dUMP and uridine), which were considered candidate gene/protein/metabolite biomarkers.

The protein encoded by *Rrm2*, called RRM2, is a ribonucleotide reductase involved in regulating DNA synthesis and repair [31]. DNA damage is a common mechanism underlying the cytotoxic effects of RRM2 [32]. This is because RRM2 can be replaced by the ribonucleotide reductase regulatory TP53 inducible subunit M2B (RRM2B), which regulates the synthesis of deoxyribonucleotide triphosphates (dNTPs) required for DNA damage repair [33]. Research has shown that RRM2 primarily affects cell proliferation and growth through activation of the Akt/mTOR signalling pathway [34].

Uric acid is the product of purine metabolism and serves as an alternative physiological substrate of myeloperoxidase. Myeloperoxidase oxidizes uric acid to uric acid free radicals and urate hydroperoxide (a strong oxidant and potential antimicrobial agent). Uric acid can significantly increase the production of superoxide [35]. Uric acid and its monoanionic urate promote oxidative damage and lead to the inactivation of enzymes sensitive to oxidative stress [36]. Uric acid oxidation products can react with glutathione, cysteine and thiol peroxidase [37]. The intracellular accumulation of uric acid at high concentrations can induce the generation of ROS and reactive nitrogen species (RNS) and activate COX-2, resulting in proinflammatory effects [38]. Based on the above findings, the increase in uric acid induced by Ni^2+^ might lead to oxidative damage.

dGMP is a type of deoxyribonucleotide that serves as the basic unit of DNA. dGMP is a small molecule composed of purine or pyrimidine bases, deoxyribose and phosphate. DNA methylation is the basis of DNA synthesis and plays a role in promoting cell growth, enhancing cell vitality and altering metabolism. Zhang *et al.* [39] reported that cell proliferation inhibition caused by guanine-rich oligonucleotides might be related to dGMP. In the present study, the level of dGMP decreased after exposure to Ni^2+^, indicating a potential association between the decrease in cell proliferation rate and dGMP.

dUMP is a substrate for thymidylate synthase, which is a rate-limiting enzyme that catalyzes the conversion of dUMP to deoxythymidine monophosphate (dTMP). dTMP is further metabolized to 2′-deoxyguanosine 5′-triphosphate (dTTP) for DNA synthesis [40]. The increase in dUMP levels after exposure to Ni^2+^ suggested inhibition of thymidylate synthase function, leading to a decrease in intracellular dTTP levels and thus affecting DNA synthesis [41].

Uridine belongs to the class of nucleoside compounds and is a critical metabolite in organisms. Uridine is an essential pyrimidine nucleoside for RNA synthesis and can be synthesized *de novo* in mammals [42]. Uridine also plays a crucial role in maintaining cell function and energy metabolism by accelerating the biosynthesis of proteins and nucleic acids, as well as energy production. Moreover, uridine and its derivatives contribute to reducing cytotoxicity and maintaining basic cellular functions based on UPase enzyme activity and ATP consumption [43, 44]. Uridine supplementation has been shown to rescue pyrimidine biosynthesis defects and has beneficial effects on mitochondrial activity. Supplementation can enhance the activity of human stem cells and promote tissue regeneration in various mammalian tissues [45]. Conversely, a decrease in uridine levels may affect mitochondrial activity, leading to a reduction in ATP synthesis and the induction of cytotoxicity.

The above analysis showed that the most critical gene/protein pair, metabolite and metabolic pathway networks can be identified only through the integrated analysis of transcriptomics, proteomics and metabolomics data. This integrative analysis further allows for a comprehensive and holistic analysis of the regulatory effects of upstream gene/protein pairs on downstream metabolites within the cell. This capability goes beyond individual omics studies, which address only a single biological layer, and combined analysis of transcriptomics and proteomics data, which is limited to upstream biological processes.

### Validation experiments for candidate gene/protein/metabolite biomarkers

The use of bioinformatics analysis can theoretically reveal the potential upstream and downstream relationships and functions of screened candidate gene/protein/metabolite biomarkers. However, the actual existence of these upstream and downstream relationships, as well as their specific roles in the interaction between biomaterials and cells, cannot be solely confirmed through theoretical analysis. Therefore, ‘dry methods’ (theoretical analysis) should be combined with ‘wet methods’ (experimental validation), and a variety of cellular/molecular biology techniques should be used to validate the expression levels and biological functions of biomarkers.

However, in the field of biomaterials, researchers have focused extensive attention on validation experiments related to the mechanisms of interactions between biomaterials and cells but have not conducted validation experiments to study cytotoxicity biomarkers of biomaterials (including expression level validation and functional validation of biomarkers). As a result, they cannot understand the characteristics and differences in validation experiments associated with these two biological experiments with distinct research objectives. Based on our previous work and the results in this article, for the first time, we summarize the applicable subjects, classifications, purposes, methods and characteristics of the validation experiments for these two types of biomics experiments in Table 5 to provide a reference for subsequent researches.

**Table 5.** The applicable subjects, classifications, purposes and different characteristics of associated technical applications in the validation experiments of biomics

No.	Applicable subjects	Classifications and purposes	Methods	Type of biomics	Type of cells
1	Study of the interaction mechanisms between biomaterials and cells	(1) Expression level validation of key genes/proteins/metabolites	qRT‒PCR, western blot, GC‒MS/LC‒MS, etc.	(1) Single omics (2) Integrative analysis of two omics (3) Integrative analysis of multiomics	Standard cells
		(2) Functional validation of important biological pathway	MTT assay, immunofluorescence staining, flow cytometry, etc.		
2	Study of cytotoxicity biomarkers of biomaterials	(1) Expression level validation of candidate biomarkers	qRT‒PCR, western blot, GC‒MS/LC‒MS, etc.	(1) Single omics (2) Integrative analysis of two omics (3) Integrative analysis of multiomics	(1) Standard cells (2) and (3): Specific cells with targeted gene silencing/overexpression
		(2) Functional validation of target biomarkers	MTT assay, immunofluorescence staining, flow cytometry, etc.	(1) Single omics (2) Integrative analysis of two omics (3) Integrative analysis of multiomics	Specific cells with targeted gene silencing/overexpression

In studies aimed at understanding the mechanisms underlying interactions between biomaterials and cells, validating the expression levels of key genes/proteins/metabolites identified through various omic experiments (single omics, integrative analysis of two omics or integrative analysis of multiple omics) will confirm the reliability of the transcriptomic, proteomic and metabolomic results. The methods employed for validation include qRT-PCR, western blotting and gas chromatography–mass spectrometry (GC–MS)/LC–MS. The purpose of validating the functions of important biological pathways identified through bioinformatics analysis is to confirm the accuracy of the bioinformatics results. The methods employed for this validation include MTT assays, immunofluorescence staining, flow cytometry, etc. The distinctive feature of these validation experiments is that they can be conducted solely with standard cells, and they have been applied widely [9, 13, 14, 25, 27, 28].

In studies aimed at identifying cytotoxicity biomarkers of biomaterials, the two kinds of validation experiments mentioned above should be included but should be specifically targeted towards the biomarkers themselves. (i) Validation of the expression levels of candidate biomarkers should be performed; for candidate biomarkers obtained through single omics methods, the validation methods are like those described above and standard cells are used in validation experiments. For candidate biomarkers obtained through the integrative analysis of two or more omics approaches, to ensure the accuracy of screening results for correlated biomarkers, validation experiments need to include specific cells. Specifically, RNAi/gene overexpression techniques were first employed to construct stable cells with targeted gene silencing or overexpression to change the expression levels of candidate gene biomarkers, and these changes were confirmed through qRT–CR. Subsequently, qRT–PCR, western blotting, GC–MS/LC–MS, etc., were utilized to validate the expression levels of candidate gene/protein/metabolite biomarkers in cells before and after exposure to biomaterials to determine the target gene/protein/metabolite biomarkers. (ii) Validation of the function of target biomarkers should be performed; to determine whether target biomarkers are detectable by single omics, integrative analysis of two omics or multiple omics approaches, functional validation experiments need to be conducted using stable cells with targeted gene silencing or overexpression. This approach is crucial for determining whether target biomarkers can be confirmed as biomarkers. However, previous studies on biomarkers of biomaterials lack this crucial step; hence, the study outcomes remain at a theoretical stage, thus limiting their guidance for practical applications. In our previous research wherein, we identified the cytotoxicity genes/protein biomarkers of Ni^2+^, we applied the technique of constructing stable cell lines with silencing/overexpression of specific target gene biomarkers for functional validation of genes/proteins in biomaterials for the first time; we identified one gene/protein biomarker that was ultimately differentially expressed in multiple Ni^2+^-treated groups [1]. In this article, a technique for constructing stable cell lines with the silencing/overexpression of target gene biomarkers was employed, and the relationships between changes in the expression levels of these genes and alterations in cell functionality were further investigated through a series of cellular methods. Thus, the gene/protein/metabolite biomarkers were confirmed; more importantly, the regulatory network from gene transcription to protein expression to metabolite alteration to cellular function could be elucidated, and the underlying mechanisms involved in Ni^2+^-induced cytotoxicity could be explored. To date, this research approach has not been reported.

### Construction of stable gene silencing/overexpression cell lines

RNAi/overexpression techniques were employed to construct stable *Rrm2*-silenced/overexpressing cell lines for three purposes: (i) to validate the correlation between the changes of candidate gene, protein and metabolite biomarkers, (ii) to validate the correlation between changes in candidate gene, protein and metabolite biomarkers and Ni^2+^ treatment, (iii) to validate the functions of biomarkers of Ni^2+^ cytotoxicity. This process helped identify correlated target gene/protein/metabolite biomarkers of Ni^2+^-induced cytotoxicity, thus reveal the underlying mechanism of these biomarkers.

**Figure 3.**
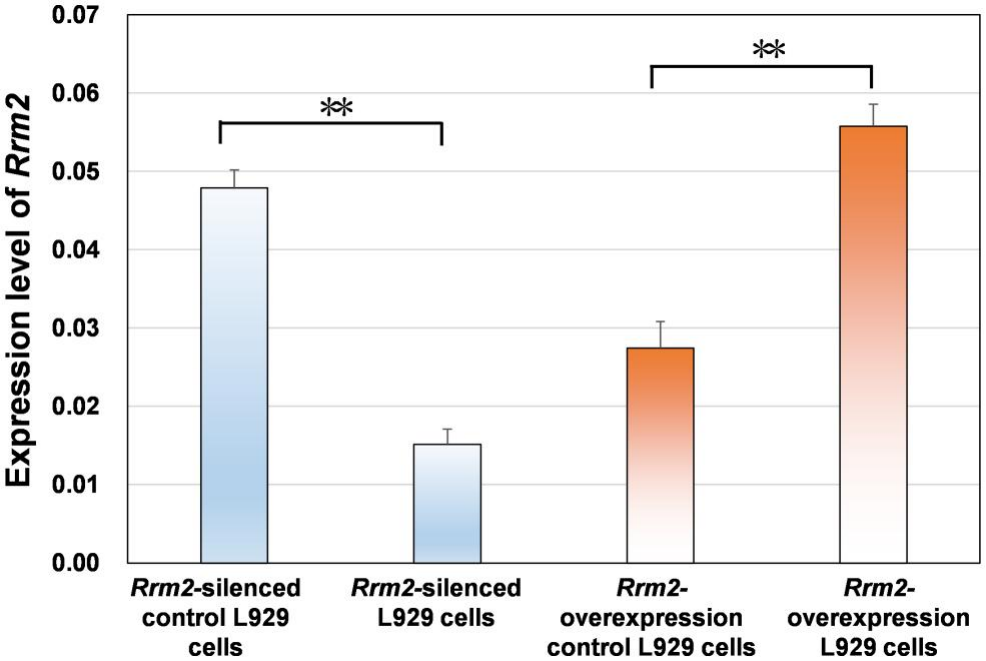
Expression level of *Rrm2* gene in four types of L929 cells.

The success of the silencing/overexpression experiments was first confirmed using qRT–PCR. The qRT–PCR results of *Rrm2* in four types of L929 cells (*Rrm2-*silenced control cells, *Rrm2-*silenced cells, *Rrm2*-overexpressing control cells and *Rrm2*-overexpressing cells) are shown in Figure 3. The expression level of *Rrm2* in the *Rrm2-*silenced cells was 68% lower than that in the *Rrm2-*silenced control cells, while in the *Rrm2*-overexpressing cells, the expression level of *Rrm2* was 103% greater than that in the *Rrm2-*overexpressing control cells. Generally, a decrease of 50% or an increase of more than twofold in the expression level of the target gene in cells after RNAi or overexpression is considered successful interference or overexpression [1]. The results in Figure 3 indicate the successful construction of *Rrm2*-silenced/overexpressing cell lines.

#### Validation of the expression levels of candidate genes/proteins/metabolites biomarkers

qRT–PCR, western blotting, and LC–MS/MS were used to validate the changes in the expression levels of the candidate *Rrm2* gene biomarker, RRM2 protein biomarker and four metabolite biomarkers (uric acid, dGMP, dUMP and uridine) before and after Ni^2+^ treatment.

##### qRT-PCR experiment

**Figure 4.**
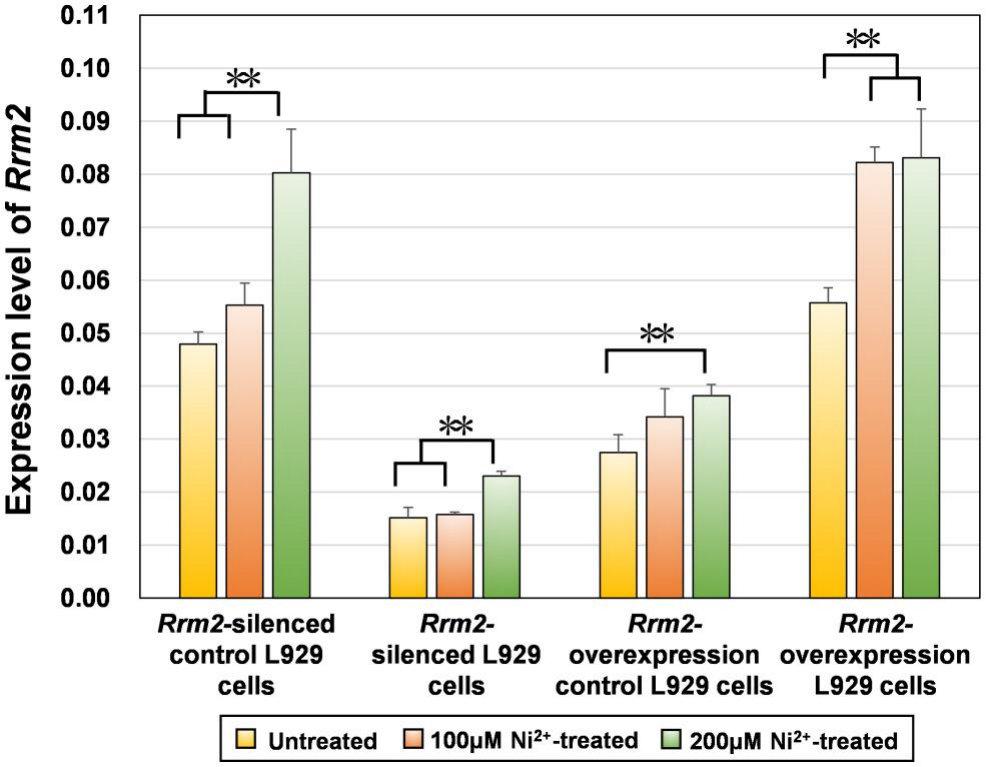
Expression level of *Rrm2* gene in untreated and Ni^2+^-treated groups in four types of L929 cells.

Figure 4 shows the expression of the *Rrm2* gene in four types of L929 cells (*Rrm2-*silenced control cells, *Rrm2-*silenced cells, *Rrm2*-overexpressing control cells and *Rrm2*-overexpressing cells) after treatment with 100 or 200 μM Ni^2+^ for 12 h. The expression levels of the *Rrm2* gene increased in the Ni^2+^-treated groups after 12 h, consistent with previous transcriptomic results [1].

##### Western blotting

**Figure 5.**
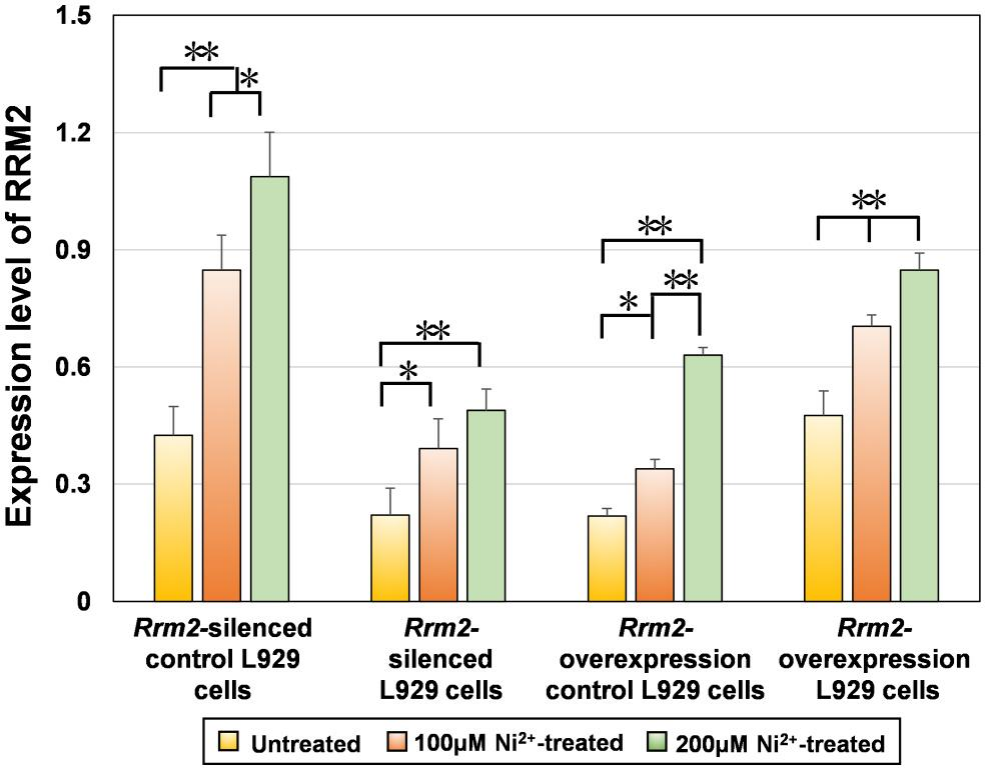
Expression level of RRM2 protein in untreated and Ni^2+^-treated groups in four types of L929 cells.

Figure 5 shows the expression of the RRM2 protein in four types of L929 cells (Rrm2-silenced control cells, Rrm2-silenced cells, Rrm2-overexpressing control cells and Rrm2-overexpressing cells) before and after treatment with 100 or 200 μM Ni^2+^ for 12 h. The semiquantitative data for the RRM2 protein are listed in Supplementary Table S8.

To determine whether changes in *Rrm2* transcription led to changes in protein levels, changes in RRM2 protein expression in four untreated groups were compared. The results revealed that the expression level of the RRM2 protein was significantly lower in the *Rrm2-*silenced cells than in the *Rrm2-*silenced control cells but was significantly greater in the *Rrm2*-overexpressing cells than in the *Rrm2*-overexpressing control cells. This indicated that a decrease or increase in *Rrm2* transcription could result in a corresponding decrease or increase in RRM2 protein expression.

Furthermore, the impact of Ni^2+^ on RRM2 protein expression was examined. After 12 h of treatment with 100 or 200 μM Ni^2+^, the expression level of the RRM2 protein increased in all four types of L929 cells, consistent with previous proteomic results [25]. This finding suggested that Ni^2+^ could influence the expression of the *Rrm2* gene, leading to changes in RRM2 protein expression levels.

##### LC-MS/MS experiment

**Figure 6.**
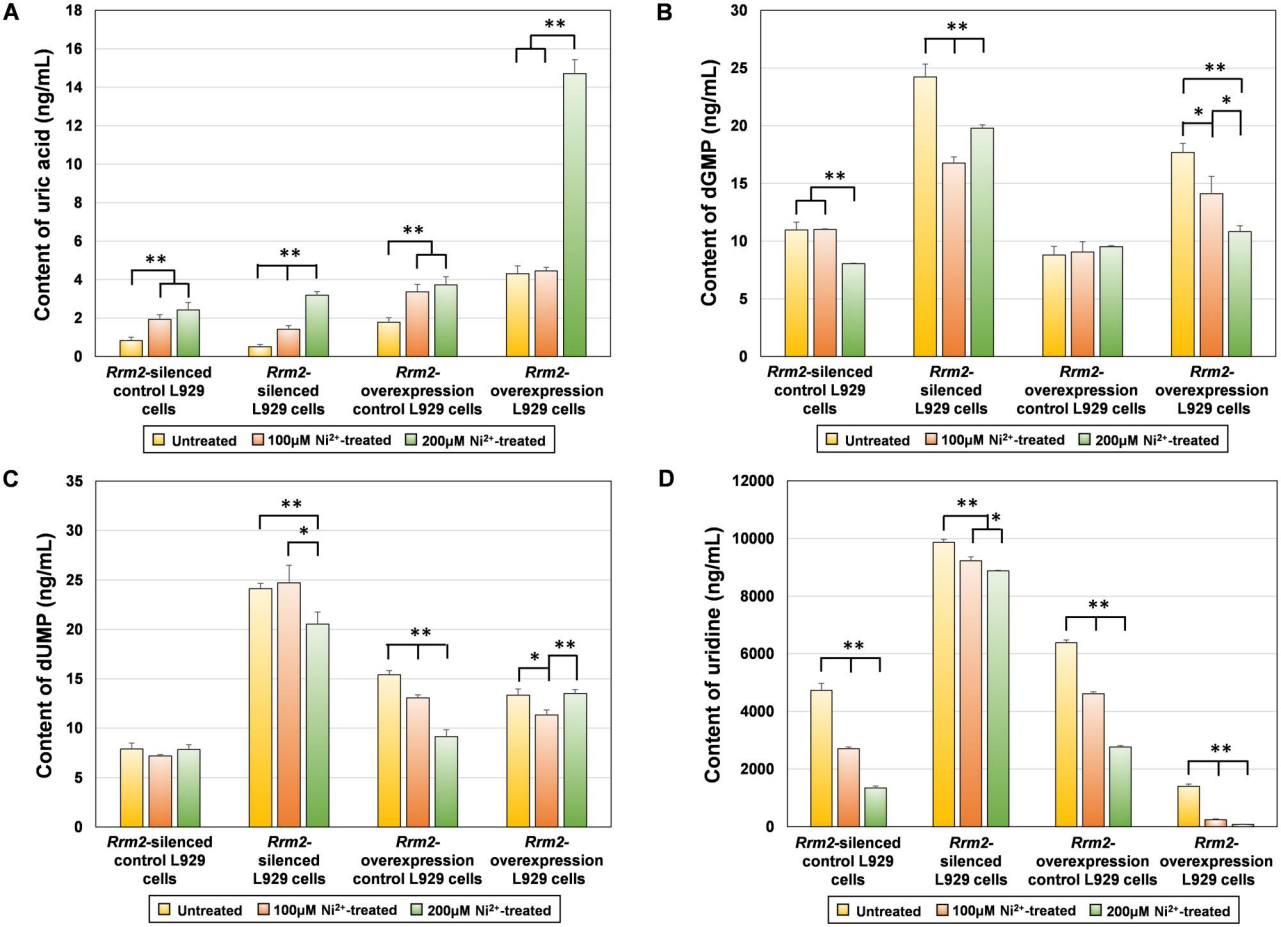
The contents of (**A**) uric acid, (**B**) dGMP, (**C**) dUMP and (**D**) uridine in untreated and Ni^2+^-treated groups in four types of L929 cells.

The contents of uric acid, dGMP, dUMP and uridine in four types of L929 cells (*Rrm2-*silenced control cells, *Rrm2-*silenced cells, *Rrm2*-overexpressing control cells and *Rrm2*-overexpressing cells) before and after 12 h of treatment with 100 or 200 μM Ni^2+^ determined via LC–MS/MS are shown in Figure 6.

To determine whether changes in *Rrm2* transcription levels lead to changes in downstream metabolite levels, changes in the levels of the four metabolites in four untreated groups were compared. As shown in Figure 6, the uric acid content was lower in *Rrm2-*silenced cells than in the *Rrm2-*silenced control cells, while the contents of dGMP, dUMP and uridine were greater than those in the *Rrm2-*silenced control cells. In the *Rrm2*-overexpressing cells, the uric acid and dGMP contents were greater than those in the *Rrm2*-overexpressing control cells, while the dUMP and uridine contents were lower than those in the *Rrm2*-overexpression control cells. This indicated that a decrease or increase in *Rrm2* transcription could result in a decrease or increase in uric acid content and an increase or decrease in dUMP and uridine contents. However, the change in dUMP levels with *Rrm2* expression was opposite to the corresponding relationship between them shown in Figure 2A. Further analysis revealed that after 12 h of Ni^2+^ treatment, the uric acid content increased and the uridine level decreased in all four types of L929 cells, indicating that Ni^2+^ affected the synthesis of these two metabolites.

The above results indicated that the target biomarkers associated with Ni^2+^-induced cytotoxicity were the *Rrm2* gene/the RRM2 protein/uric acid and uridine. The established regulatory network included *Rrm2* gene transcription changes, changes in RRM2 protein expression and changes in uric acid and uridine levels.

#### Functional validation of target gene/protein/metabolite biomarkers

Bioinformatics analysis and literature review of the target gene (*Rrm2*)/protein (RRM2)/metabolite biomarkers (uric acid and uridine) (‘Screening of candidate gene/protein/metabolite biomarkers’ section) of Ni^2+^ showed that their functions were primarily associated with DNA synthesis, cell proliferation, oxidative stress and ATP synthesis. Therefore, the following four experiments were conducted using the *Rrm2* silencing/overexpression stable cell lines constructed in ‘Construction of stable gene silencing/overexpression cell lines’ section to validate the role of the target gene/protein/metabolite biomarkers in Ni^2+^-induced cytotoxicity.

##### Cell cycle analysis

**Figure 7.**
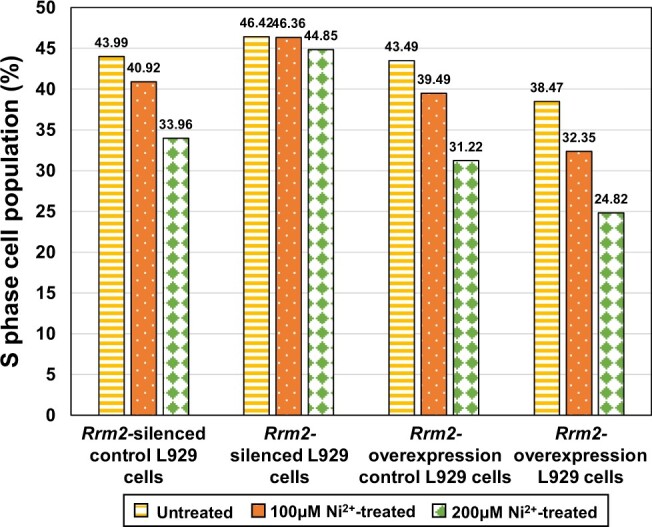
S phase cell population in untreated and Ni^2+^-treated groups for four types of L929 cells.

Flow cytometry analysis results are shown in Supplementary Figure S6. The cell cycle consists of several phases, including the G1 phase, where the cell size increases; the S phase, where new DNA is synthesized; the G2 phase, where the cell further grows; and the M phase, where mitosis occurs and the cell eventually divides. Since *Rrm2* affects DNA synthesis, in this study, we focused on changes in S phase cells. Figure 7 shows that the S phase cell population of four types of L929 cells (*Rrm2-*silenced control cells, *Rrm2-*silenced cells, *Rrm2*-overexpressing control cells and *Rrm2*-overexpressing cells) in each Ni^2+^-treated group was lower than that in the untreated group, with the 200 μM Ni^2+^-treated group showing the lowest population. These results indicated that Ni^2+^ upregulated *Rrm2* gene and RRM2 protein expression (Figures 4 and 5) so that inhibited DNA synthesis, and the higher the concentration was, the greater the impact.

##### Cell proliferation rate determination

**Figure 8.**
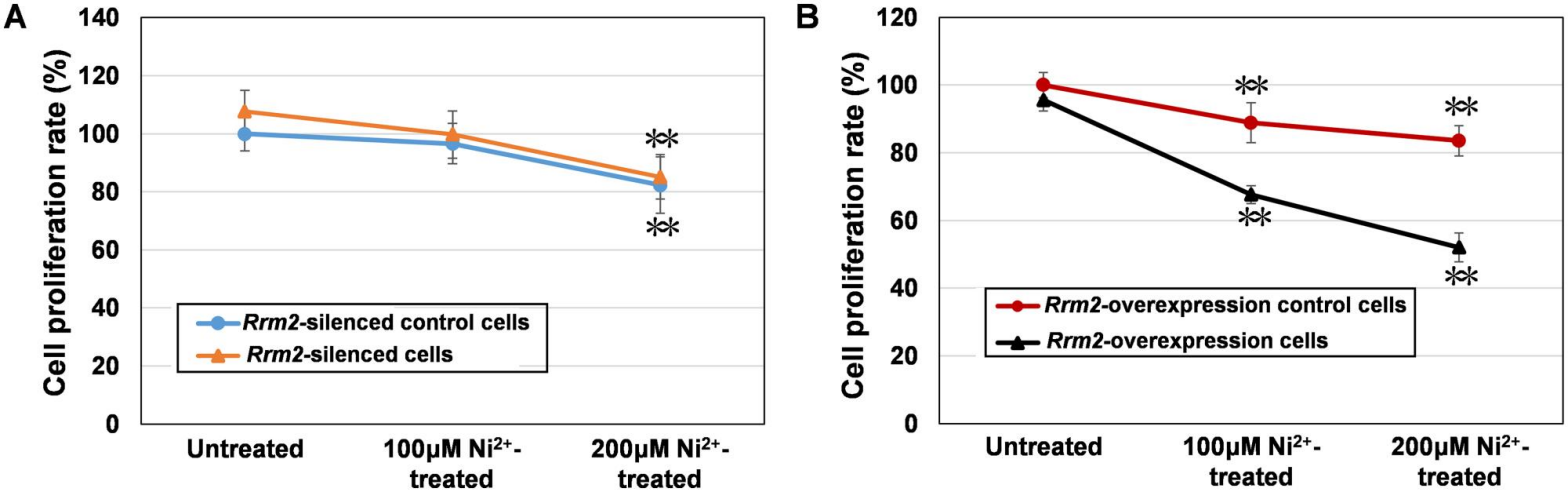
The cell proliferation rate in the untreated and Ni^2+^-treated groups. (**A**) Proliferation rate of *Rrm2-*silenced control cells and *Rrm2-*silenced cells. (**B**) Proliferation rate of *Rrm2*-overexpressing control cells and *Rrm2*-overexpressing cells.

Figure 8 shows the cell proliferation rates of four types of L929 cells (*Rrm2-*silenced control cells, *Rrm2-*silenced cells, *Rrm2*-overexpressing control cells and *Rrm2*-overexpressing cells) after 12 h of treatment with 100 or 200 μM Ni^2+^. The cell proliferation rates in the Ni^2+^-treated groups were lower than those in the untreated group for the same cell type, indicating that Ni^2+^ could upregulate *Rrm2* gene and RRM2 protein expression (Figures 4 and 5) and then suppress cell proliferation.

##### Oxidative stress analysis

**Figure 9.**
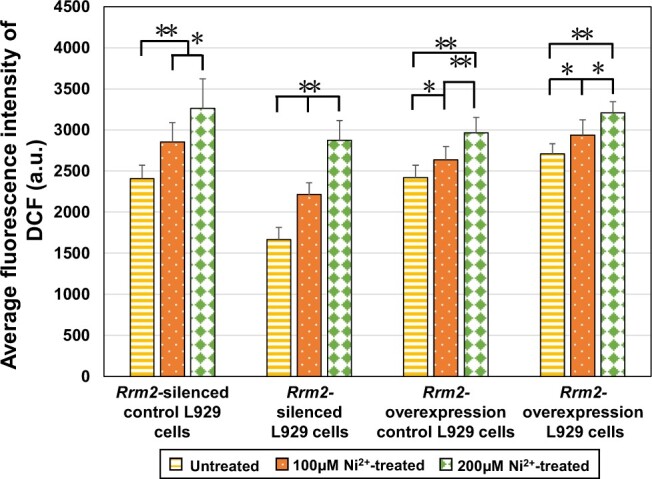
ROS level in untreated and Ni^2+^-treated groups in four types of L929 cells.

Figure 9 displays the average fluorescence intensity of DCF in four types of L929 cells (*Rrm2-*silenced control cells, *Rrm2-*silenced cells, *Rrm2*-overexpressing control cells and *Rrm2*-overexpressing cells) after 12 h of treatment with 100 or 200 μM Ni^2+^. A higher fluorescence intensity indicates a greater degree of oxidative stress. The average fluorescence intensity of DCF in each Ni^2+^-treated group showed a significant or highly significant increase compared to that in the untreated group of the same cell type, demonstrating that Ni^2+^ upregulated *Rrm2* gene and RRM2 protein expression (Figures 4 and 5) and then increased uric acid synthesis (Figure 6) which led to an increase of oxidative stress level in the cells.

##### ATP content measurement

**Figure 10.**
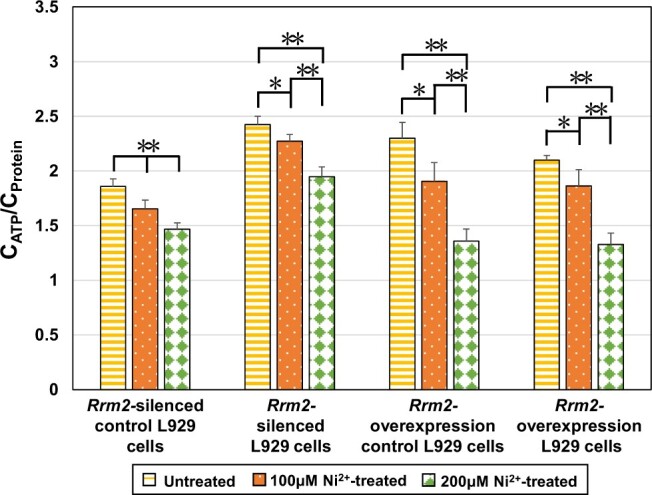
ATP content in untreated and Ni^2+^-treated groups in four types of L929 cells.

Figure 10 shows the ATP content in four types of L929 cells (*Rrm2-*silenced control cells, *Rrm2-*silenced cells, *Rrm2*-overexpressing control cells and *Rrm2*-overexpressing cells) after 12 h of treatment with 100 or 200 μM Ni^2+^. The ATP content in each Ni^2+^-treated group showed a significant or highly significant decrease compared to that in the untreated group of the same cell type, indicating that Ni^2+^ upregulated *Rrm2* gene and RRM2 protein expression (Figures 4 and 5) and then decreased uridine synthesis (Figure 6) which result in a decrease of ATP synthesis.

**Figure 11.**
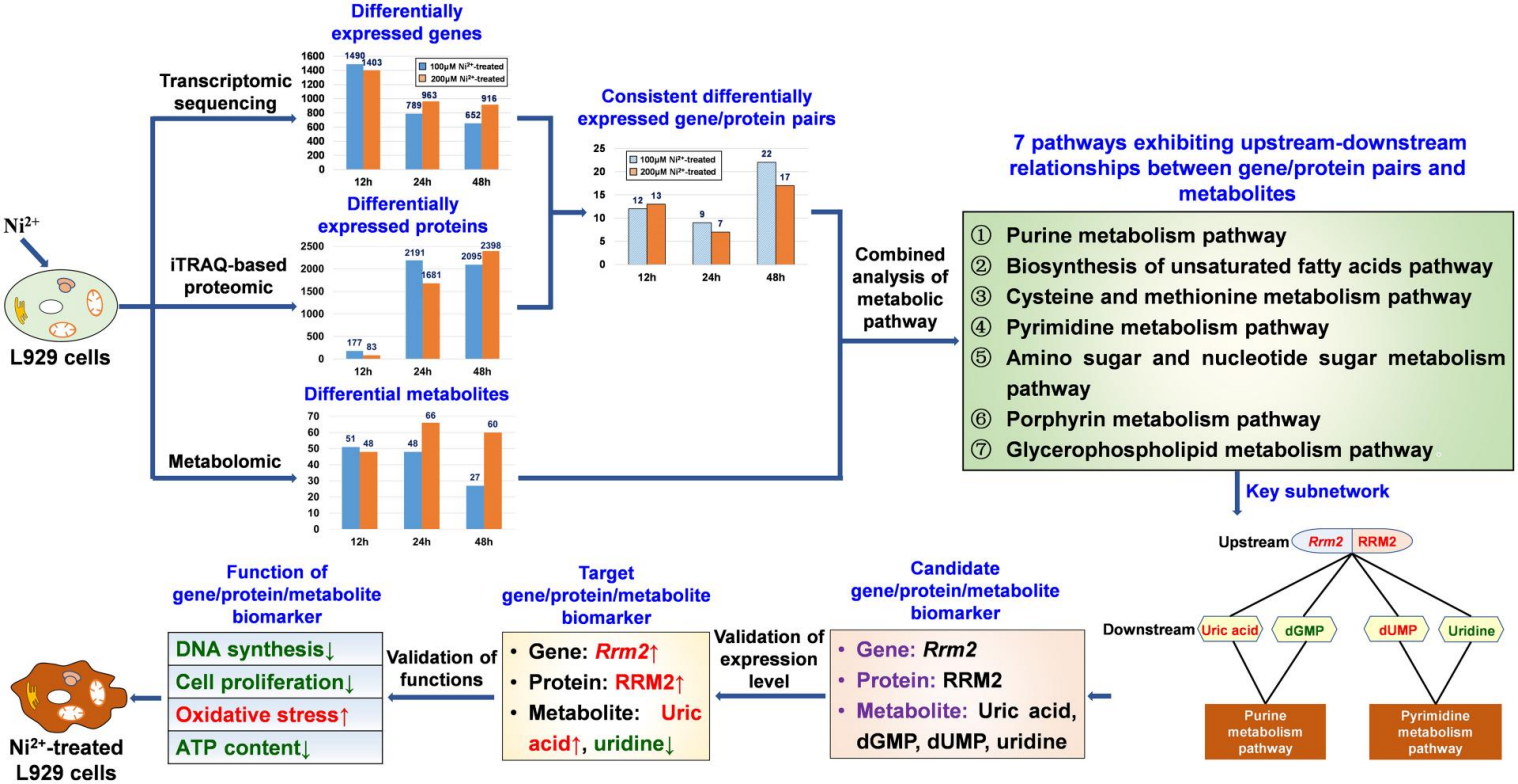
The role of gene/protein/metabolite biomarkers in the interaction between Ni^2+^ and L929 cells.

So the gene, protein and metabolite biomarkers associated with Ni^2+^ cytotoxicity were *Rrm2* gene, RRM2 protein, uric acid and uridine. The mechanism of action of Ni^2+^-induced cytotoxicity was as follows: Ni^2+^ influenced the expression of the *Rrm2* gene biomarker, which subsequently affected the expression of the RRM2 protein biomarker, leading to alterations in uric acid and uridine metabolite biomarkers. These changes further impacted DNA synthesis, cell proliferation, intracellular ROS levels and ATP content (Figure 11).

A comparison of the Ni^2+^ biomarker data reported in the literature and that collected by our research group is summarized in Table 6. The data collected in this article differs from other research in terms of the methods and the selection criteria for gene/protein/metabolite biomarkers. This study demonstrated innovations in terms of the approach, methods and results.

**Table 6.** Comparison of Ni^2+^ biomarker studies reported in the literature and this article

No.	Literature	Method	Screened potential Ni^2+^ biomarkers (Results)	Selection criteria (Research approach)
1	Acevedo *et al.* [18]	2D gel electrophoresis, amino acid sequencing	ANXA2, PGK	Differentially expressed proteins
2	Clemens *et al.* [19]	RT‒PCR, northern blotting, Southern blotting, western blotting	*Ect* gene/protein	Derived from other literature and their research in earlier phases
3	Kim *et al.* [20]	Gene chip, PCR	*Nrf2* gene	Derived from other literature and their research in earlier phases
4	Kim *et al.* [21]	Gene chip	11 genes	*Trr1:* Derived from other literature Other 10 genes: Showed differential expression after *Trr1* knockout.
5	Kwon *et al.* [22]	Gene chip, functional proteomics	26 genes 16 proteins	Gene: differentially expressed genes. Protein: differentially expressed proteins and network analysis
6	Our previously published paper [1]	Transcriptomic sequencing, proteomics	Gene/protein: *Uqcrb*/UQCRB	(1) Integrative screening of two omics data (2) Biological pathway analysis (3) Expression levels validation of target biomarkers and function
7	This article	Transcriptomics, proteomics, metabolomics	Gene: *Rrm2* Protein: RRM2 Metabolite: Uric acid, uridine	(1) Integrative analysis of three omics data (2) Metabolic pathway analysis (3) Expression level validation and function validation

## Conclusion

In this article, for the first time, we conducted an integrative analysis of transcriptomic, proteomic and metabolomic data and investigated the complete biological process from gene transcription to protein expression to metabolite alteration, leading to the identification of candidate gene/protein/metabolite biomarkers associated with Ni^2+^-induced cytotoxicity. Subsequently, by validating their relationships at the expression level, target gene/protein/metabolite biomarkers were identified and a comprehensive regulatory network related to Ni^2+^-induced cytotoxicity, encompassing gene transcription, protein expression and metabolite alteration, was constructed. Finally, through functional validation of the target biomarkers, the gene (*Rrm2*), protein (RRM2) and metabolite (uric acid and uridine) biomarkers were confirmed. Mechanistically, in Ni^2+^-induced cytotoxicity, Ni^2+^ affected the expression of the gene biomarker *Rrm2*, the protein biomarker RRM2 and the metabolite biomarkers uric acid and uridine in a top-down manner, ultimately inhibiting DNA synthesis, suppressing cell proliferation, increasing intracellular ROS levels and reducing ATP content. This article not only broadens our understanding of the mechanisms underlying the interaction of Ni^2+^ with cells but also explores an effective new approach for the identification of cytotoxic biomarkers of biomaterials based on multi-omics technologies, bioinformatics analysis and biological validation. This technological approach and these findings have not been previously reported.

## Supplementary Material

rbae079_Supplementary_Data
