# Supplementary material for: Combination of gene/protein and metabolite multiomics to reveal biomarkers of nickel ion cytotoxicity and the underlying mechanism
Source: Regen Biomater. 2024 Jun 29;11:rbae079. doi: 10.1093/rb/rbae079 (PMC11254314; doi:10.1093/rb/rbae079)
Supplement: rbae079_Supplementary_Data [file rbae079_supplementary_data.docx]

**Supplementary Table S1:** Consistently differentially expressed gene/protein pairs.

| **Group** | **No.** | **Official gene symbol** | **Fold Change of gene^*^** | **Fold Change of protein^#^** |
| --- | --- | --- | --- | --- |
| 100µM Ni^2+^-12h | 1 | RRM2 | 3.679 | 1.360 |
|  | 2 | MKI67 | 2.701 | 1.203 |
|  | 3 | SLC2A1 | 2.631 | 1.317 |
|  | 4 | EGLN1 | 2.345 | 1.412 |
|  | 5 | PPL | 2.253 | 1.567 |
|  | 6 | LBH | 0.480 | 0.772 |
|  | 7 | STARD5 | 0.451 | 0.706 |
|  | 8 | ACOT2 | 0.423 | 0.751 |
|  | 9 | FAH | 0.332 | 0.779 |
|  | 10 | GNPNAT1 | 0.323 | 0.649 |
|  | 11 | HYOU1 | 0.252 | 0.832 |
|  | 12 | ERO1LB | 0.228 | 0.820 |
| 100µM Ni^2+^-24h | 1 | LRRC8C | 0.493 | 0.482 |
|  | 2 | DNAJC3 | 0.488 | 0.494 |
|  | 3 | FAH | 0.475 | 0.557 |
|  | 4 | PDIA4 | 0.451 | 0.469 |
|  | 5 | MAT2A | 0.437 | 0.507 |
|  | 6 | CRELD2 | 0.426 | 0.613 |
|  | 7 | HYOU1 | 0.403 | 0.477 |
|  | 8 | EXOSC6 | 0.387 | 0.646 |
|  | 9 | ERO1LB | 0.383 | 0.512 |
| 100µM Ni^2+^-48h | 1 | NDRG1 | 3.975 | 1.516 |
|  | 2 | NDUFA4L2 | 3.387 | 1.584 |
|  | 3 | SELENBP1 | 3.208 | 1.334 |
|  | 4 | ERO1L | 2.650 | 1.222 |
|  | 5 | PPL | 2.176 | 1.389 |
|  | 6 | ACOT2 | 0.491 | 0.636 |
|  | 7 | DDX21 | 0.487 | 0.596 |
|  | 8 | DNAJB11 | 0.486 | 0.727 |
|  | 9 | FAH | 0.479 | 0.692 |
|  | 10 | NOLC1 | 0.469 | 0.642 |
|  | 11 | CSF1 | 0.465 | 0.551 |
|  | 12 | BYSL | 0.456 | 0.615 |
|  | 13 | HYOU1 | 0.446 | 0.716 |
|  | 14 | RRS1 | 0.444 | 0.643 |
|  | 15 | GNPNAT1 | 0.438 | 0.534 |
|  | 16 | EXOSC6 | 0.436 | 0.688 |
|  | 17 | RRP12 | 0.418 | 0.713 |
|  | 18 | CRELD2 | 0.408 | 0.674 |
|  | 19 | CCDC86 | 0.401 | 0.716 |
|  | 20 | MAT2A | 0.372 | 0.734 |
|  | 21 | 2210010C04Rik | 0.357 | 0.762 |
|  | 22 | CTH | 0.339 | 0.528 |
| 200µM Ni^2+^-12h | 1 | RRM2 | 3.758 | 1.456 |
|  | 2 | SLC2A1 | 3.555 | 1.519 |
|  | 3 | EGLN1 | 3.174 | 1.633 |
|  | 4 | NDRG1 | 2.798 | 1.586 |
|  | 5 | UBE2C | 2.326 | 1.438 |
|  | 6 | PMF1 | 2.276 | 1.491 |
|  | 7 | SELENBP1 | 2.025 | 1.330 |
|  | 8 | Uqcrb | 0.495 | 0.813 |
|  | 9 | CP | 0.479 | 0.606 |
|  | 10 | ACOT2 | 0.465 | 0.805 |
|  | 11 | LBH | 0.459 | 0.776 |
|  | 12 | GNPNAT1 | 0.320 | 0.747 |
|  | 13 | ERO1LB | 0.290 | 0.829 |
| 200µM Ni^2+^-24h | 1 | MUT | 0.494 | 0.483 |
|  | 2 | LBH | 0.491 | 0.705 |
|  | 3 | Uqcrb | 0.457 | 0.443 |
|  | 4 | CSF1 | 0.385 | 0.313 |
|  | 5 | PSPC1 | 0.366 | 0.711 |
|  | 6 | GPD1 | 0.360 | 0.509 |
|  | 7 | LRRC8C | 0.352 | 0.535 |
| 200µM Ni^2+^-48h | 1 | NDRG1 | 6.284 | 1.771 |
|  | 2 | SELENBP1 | 4.559 | 1.233 |
|  | 3 | ERO1L | 3.751 | 1.230 |
|  | 4 | CALU | 0.495 | 0.588 |
|  | 5 | LBH | 0.494 | 0.474 |
|  | 6 | NDC1 | 0.487 | 0.687 |
|  | 7 | TRIP13 | 0.477 | 0.566 |
|  | 8 | ACSF2 | 0.472 | 0.529 |
|  | 9 | ADCK1 | 0.472 | 0.471 |
|  | 10 | GPD1 | 0.441 | 0.468 |
|  | 11 | MCM4 | 0.430 | 0.558 |
|  | 12 | NQO2 | 0.424 | 0.499 |
|  | 13 | LRRC8C | 0.380 | 0.522 |
|  | 14 | GEMIN4 | 0.373 | 0.643 |
|  | 15 | CSF1 | 0.358 | 0.400 |
|  | 16 | RARRES2 | 0.318 | 0.563 |
|  | 17 | MAT2A | 0.293 | 0.692 |

**^*^**Fold Change of gene>2 means gene up-regulated, fold change of gene<0.5 means gene down-regulated.

^#^Fold Change of protein>1.2 means protein up-regulated, fold change of gene<0.833 means protein down-regulated.

**Supplementary Table S2:** Differential metabolites in 100µM Ni^2+^-12h group.

| **No.** | **Name** | **VIP** | **mz** | **RT(min)** | **ttest** | **Log_2_FC*** |
| --- | --- | --- | --- | --- | --- | --- |
| 1 | PA(40:4) | 1.707 | 753.56 | 9.71 | 0.00 | 1.61 |
| 2 | Palmitic acid | 1.052 | 255.2321 | 8.16 | 0.02 | 1.48 |
| 3 | Guanosine triphosphate adenosine | 1.683 | 773.06 | 10.01 | 0.00 | 1.44 |
| 4 | PC(42:6) | 1.672 | 862.64 | 10.38 | 0.00 | 1.41 |
| 5 | NADH | 1.874 | 666.15 | 11.96 | 0.01 | 1.30 |
| 6 | GDP-glucose | 1.671 | 606.09 | 11.21 | 0.00 | 1.28 |
| 7 | Oleic Acid | 1.159 | 281.25 | 8.4 | 0.00 | 1.24 |
| 8 | ADP-L-glycero-D-manno-heptose | 1.600 | 620.77 | 11.6 | 0.00 | 1.22 |
| 9 | PA(33:2) | 1.732 | 659.47 | 11.71 | 0.01 | 1.19 |
| 10 | PC(32:1) | 1.187 | 732.57 | 9.5 | 0.00 | 1.03 |
| 11 | PC(38:8) | 1.301 | 800.54 | 11.82 | 0.00 | 1.03 |
| 12 | Uric acid | 1.014 | 169.04 | 0.93 | 0.01 | 0.98 |
| 13 | PC(38:5) | 1.288 | 806.59 | 11.21 | 0.00 | 0.84 |
| 14 | PGA1 methyl ester | 1.187 | 349.24 | 8.4 | 0.00 | 0.83 |
| 15 | PC(40:10) | 1.054 | 824.54 | 11.49 | 0.01 | 0.79 |
| 16 | PC(33:3) | 1.316 | 740.52 | 11.85 | 0.01 | 0.78 |
| 17 | PC(35:5) | 1.092 | 764.53 | 11.47 | 0.01 | 0.77 |
| 18 | PC(32:0) | 1.297 | 734.57 | 9.52 | 0.00 | 0.73 |
| 19 | PC(33:1) | 1.133 | 744.56 | 11.24 | 0.01 | 0.72 |
| 20 | L-Ornithine | 1.522 | 131.08 | 0.74 | 0.00 | 0.71 |
| 21 | PC(38:6) | 1.145 | 804.58 | 11.21 | 0.01 | 0.69 |
| 22 | Sphinganine | 1.152 | 302.30 | 3.73 | 0.01 | 0.60 |
| 23 | Nα-Acetyl-L-glutamine | 1.537 | 187.07 | 0.75 | 0.00 | 0.41 |
| 24 | Carnitine | 1.511 | 160.10 | 0.74 | 0.00 | 0.33 |
| 25 | L-Alanine | 1.554 | 88.04 | 0.75 | 0.00 | 0.32 |
| 26 | PC(34:1) | 1.068 | 760.58 | 9.47 | 0.02 | 0.28 |
| 27 | L-Lysine | 1.508 | 145.10 | 0.76 | 0.00 | 0.27 |
| 28 | Acetamide | 1.177 | 60.04 | 11.93 | 0.00 | -0.09 |
| 29 | Calcium L-aspartate | 1.622 | 171.9898 | 12.17 | 0.004 | -0.132 |
| 30 | 3-(Hydrohydroxyphosphoryl)pyruvate | 2.113 | 152.9946 | 12.28 | 0 | -0.257 |
| 31 | PC(40:9) | 1.043 | 828.55 | 11.89 | 0.02 | -0.29 |
| 32 | Taurocholic acid 3-sulfate | 1.244 | 596.26 | 12.08 | 0.01 | -0.29 |
| 33 | PC(40:8) | 1.158 | 830.57 | 11.92 | 0.00 | -0.36 |
| 34 | Sphingosine 1-phosphate | 1.041 | 380.26 | 3.83 | 0.01 | -0.38 |
| 35 | Acetyl phosphate | 1.534 | 141.00 | 9.51 | 0.01 | -0.49 |
| 36 | LysoPE(20:2) | 1.080 | 504.31 | 4.22 | 0.00 | -0.54 |
| 37 | oxyquinoline | 1.140 | 146.06 | 1.65 | 0.00 | -0.55 |
| 38 | PC(20:4) | 1.101 | 544.3385 | 4.16 | 0.01 | -0.65 |
| 39 | PC(36:1) | 1.092 | 788.62 | 11.98 | 0.09 | -0.65 |
| 40 | Linoleic acid | 1.215 | 279.23 | 4.18 | 0.01 | -0.66 |
| 41 | Phosphohydroxypyruvic acid | 2.103 | 184.99 | 9.51 | 0.01 | -0.68 |
| 42 | LysoPC(20:3) | 1.176 | 546.35 | 4.45 | 0.01 | -0.72 |
| 43 | PC(18:1) | 1.297 | 522.35 | 4.75 | 0.01 | -0.72 |
| 44 | PC(20:2) | 1.094 | 548.37 | 5 | 0.01 | -0.74 |
| 45 | Deoxycholic acid 3-glucuronide | 1.187 | 569.34 | 4.12 | 0.00 | -0.77 |
| 46 | PC(22:6) | 1.185 | 568.34 | 4.12 | 0.01 | -0.78 |
| 47 | PC(20:1) | 1.012 | 550.39 | 5.87 | 0.01 | -0.93 |
| 48 | N-Acetyl-DL-methionine | 1.261 | 192.07 | 1.44 | 0.00 | -0.95 |
| 49 | LysoPE(18:2) | 1.594 | 478.30 | 4.17 | 0.01 | -1.02 |
| 50 | PC(18:2) | 1.723 | 520.34 | 4.18 | 0.01 | -1.21 |
| 51 | PC(24:0) | 1.615 | 622.45 | 8.62 | 0.01 | -1.55 |

Log_2_FC>0, content of metabolite increased, Log_2_FC<0, content of metabolite decreased.

**Supplementary Table S3:** Differential metabolites in 100µM Ni^2+^-24h group.

| **No.** | **Name** | **VIP** | **mz** | **RT(min)** | **ttest** | **Log_2_FC** |
| --- | --- | --- | --- | --- | --- | --- |
| 1 | PC(20:0) | 2.026 | 550.39 | 15.44 | 0.00 | 1.34 |
| 2 | keto myristic acid | 1.512 | 241.18 | 13.14 | 0.01 | 1.21 |
| 3 | hydroxy-decanoic acid | 1.532 | 187.13 | 10.15 | 0.01 | 1.14 |
| 4 | PC(O-18:0) | 1.453 | 508.38 | 14.37 | 0.00 | 1.00 |
| 5 | Dodecanedioic acid | 1.236 | 229.14 | 9.76 | 0.03 | 0.86 |
| 6 | PE(18:2) | 1.625 | 476.28 | 12.72 | 0.00 | 0.79 |
| 7 | PC(20:1) | 1.522 | 548.37 | 14.4 | 0.00 | 0.79 |
| 8 | PC(17:2)/PE(20:2) | 1.768 | 504.31 | 13.04 | 0.00 | 0.68 |
| 9 | PC(18:1) | 1.275 | 520.34 | 13.07 | 0.00 | 0.63 |
| 10 | PC(17:1)/PE(20:1) | 1.924 | 506.33 | 13.95 | 0.00 | 0.63 |
| 11 | PC(18:0) | 1.259 | 522.35 | 13.07 | 0.01 | 0.61 |
| 12 | PS(21:0) | 1.731 | 566.35 | 13.95 | 0.00 | 0.60 |
| 13 | Sphingosine 1-phosphate | 1.792 | 378.24 | 11.96 | 0.00 | 0.57 |
| 14 | PE(18:0)/PC(15:0) | 1.702 | 480.31 | 13.53 | 0.00 | 0.44 |
| 15 | PC(20:2) | 1.141 | 546.35 | 15.12 | 0.01 | 0.42 |
| 16 | Sphinganine-phosphate | 1.056 | 380.26 | 12 | 0.01 | 0.39 |
| 17 | PE(20:4) | 1.173 | 500.28 | 12.8 | 0.05 | 0.39 |
| 18 | L-Glutamine | 1.624 | 145.06 | 0.76 | 0.01 | 0.38 |
| 19 | Oxaloglutarate | 1.392 | 203.02 | 0.75 | 0.02 | 0.38 |
| 20 | 1-Linoleoylglycerophosphocholine | 1.090 | 518.32 | 13.56 | 0.02 | 0.28 |
| 21 | TG(59:9) | 1.327 | 941.79 | 0.69 | 0.02 | -0.20 |
| 22 | TG(55:2) | 1.185 | 899.80 | 0.69 | 0.04 | -0.25 |
| 23 | Vanilpyruvic acid | 1.213 | 209.05 | 5.86 | 0.04 | -0.25 |
| 24 | TG(51:0) | 1.312 | 847.79 | 0.69 | 0.02 | -0.26 |
| 25 | Methylselenocysteine Se-oxide | 1.525 | 197.96 | 0.65 | 0.01 | -0.27 |
| 26 | 3-Sulfinylpyruvic acid | 1.017 | 152.98 | 0.65 | 0.02 | -0.28 |
| 27 | L-Arginine phosphate | 1.243 | 255.09 | 6.45 | 0.00 | -0.32 |
| 28 | Dihydroceramide | 1.064 | 328.29 | 15.38 | 0.01 | -0.34 |
| 29 | Metyrosine | 1.533 | 194.08 | 12.02 | 0.00 | -0.34 |
| 30 | Uridine | 1.658 | 243.06 | 1.14 | 0.00 | -0.39 |
| 31 | Sphinganine | 1.279 | 300.29 | 13.8 | 0.00 | -0.40 |
| 32 | Glucose | 1.304 | 179.06 | 0.75 | 0.02 | -0.40 |
| 33 | Stearamide | 1.362 | 282.28 | 13.8 | 0.00 | -0.45 |
| 34 | Deamino-α-keto-demethylphosphinothricin | 1.222 | 165.00 | 0.75 | 0.04 | -0.50 |
| 35 | Hydroxylysine | 1.518 | 161.10 | 14.3 | 0.00 | -0.58 |
| 36 | N-Methylethanolamine phosphate | 1.121 | 156.04 | 0.75 | 0.01 | -0.64 |
| 37 | Succinic acid semialdehyde | 1.171 | 101.02 | 0.74 | 0.05 | -0.67 |
| 38 | Indolelactic acid | 1.227 | 204.07 | 6.64 | 0.04 | -0.71 |
| 39 | Phenylalanine | 1.527 | 166.09 | 2.22 | 0.00 | -0.71 |
| 40 | 3-Dehydroquinic acid | 1.757 | 189.04 | 1.26 | 0.00 | -0.72 |
| 41 | Creatinine | 1.255 | 114.07 | 0.77 | 0.00 | -0.73 |
| 42 | hydroxy palmitic acid | 1.646 | 271.23 | 13.9 | 0.00 | -0.77 |
| 43 | Pyroglutamic acid | 1.014 | 130.05 | 1.14 | 0.02 | -0.85 |
| 44 | Uric acid | 1.284 | 169.04 | 1.127319 | 0.00 | -1.11 |
| 45 | Trigonellinamide | 1.015 | 137.07 | 0.76 | 0.02 | -1.29 |
| 46 | N-Methyl-2-pyridone-5-carboxamide | 1.223 | 153.07 | 1.42 | 0.00 | -1.42 |
| 47 | Homovanillic acid | 1.228 | 181.05 | 4.24 | 0.04 | -1.80 |
| 48 | Acetyl-L-tyrosine | 1.473 | 222.08 | 4.45 | 0.01 | -3.25 |

**Supplementary Table S4:** Differential metabolites in 100µM Ni^2+^-48h group.

| **No.** | **Name** | **VIP** | **mz** | **RT(min)** | **ttest** | **Log_2_FC** |
| --- | --- | --- | --- | --- | --- | --- |
| 1 | L-Ascorbate 6-phosphate | 1.596 | 256.00 | 5.47 | 0.02 | 2.82 |
| 2 | Hypoxanthine | 2.024 | 136.04 | 0.96 | 0.00 | 1.96 |
| 3 | Sedoheptulose 7-phosphate | 2.496 | 290.05 | 8.66 | 0.00 | 1.30 |
| 4 | 7-Oxo-11-dodecenoic acid | 2.458 | 212.14 | 8.75 | 0.01 | 1.21 |
| 5 | progesterone | 3.036 | 314.22 | 15.23 | 0.00 | 1.13 |
| 6 | PC(21:0) | 2.184 | 579.40 | 13.66 | 0.00 | 1.13 |
| 7 | Chenodeoxycholic acid 3-sulfate | 2.981 | 472.26 | 11.73 | 0.01 | 0.95 |
| 8 | ferulic acid | 2.575 | 194.06 | 8.65 | 0.00 | 0.92 |
| 9 | Leukotriene C4 | 1.694 | 625.31 | 13.65 | 0.02 | 0.85 |
| 10 | Adrenosterone | 1.455 | 300.17 | 12.54 | 0.05 | 0.65 |
| 11 | 4,5-dehydro Docosahexaenoic Acid | 2.194 | 326.22 | 15.45 | 0.00 | 0.62 |
| 12 | Hexadecanedioic acid | 1.665 | 286.21 | 15.49 | 0.02 | 0.41 |
| 13 | Palmitoyl-L-carnitine | 1.443 | 399.33 | 13.21 | 0.06 | 0.40 |
| 14 | L-Carnitine | 1.512 | 161.10 | 0.65 | 0.04 | 0.38 |
| 15 | D-myo-Inositol-1,3,4,5-tetraphosphate | 1.186 | 499.92 | 0.57 | 0.10 | 0.28 |
| 16 | Uridine | 1.586 | 244.07 | 10.74 | 0.03 | 0.25 |
| 17 | Glycerophospho-N-Arachidonoyl Ethanolamine | 1.428 | 501.27 | 11.73 | 0.05 | -0.23 |
| 18 | Galactitol 1-phosphate | 1.945 | 262.05 | 10.09 | 0.02 | -0.31 |
| 19 | Tetradecanedioic acid | 2.962 | 258.18 | 14.1 | 0.01 | -0.46 |
| 20 | Uric acid | 1.852 | 168.03 | 0.96 | 0.01 | -0.46 |
| 21 | Androstenedione | 1.802 | 286.19 | 13.2 | 0.02 | -0.51 |
| 22 | Lipoxin C4 | 1.532 | 641.30 | 12.81 | 0.04 | -0.63 |
| 23 | PC(19:0) | 1.572 | 537.38 | 15.52 | 0.03 | -0.63 |
| 24 | PC(20:0) | 1.302 | 551.39 | 15.97 | 0.08 | -0.68 |
| 25 | N-methyl arachidonoyl amine | 1.511 | 317.2727 | 10.4 | 0.05 | -0.71 |
| 26 | 5-HETE | 2.692 | 320.24 | 13.3 | 0.01 | -0.76 |
| 27 | 12-amino-dodecanoic acid | 2.134 | 215.19 | 7.05 | 0.00 | -1.00 |

**Supplementary Table S5:** Differential metabolites in 200µM Ni^2+^-12h group.

| **No.** | **Name** | **VIP** | **mz** | **RT(min)** | **ttest** | **Log_2_FC** |
| --- | --- | --- | --- | --- | --- | --- |
| 1 | 1-Linoleoylglycerophosphocholine | 1.871 | 519.33 | 5.85 | 0.00 | 8.42 |
| 2 | 3-Furoic acid | 1.557 | 112.02 | 0.92 | 0.00 | 5.65 |
| 3 | Sebacic acid | 1.384 | 202.12 | 7.78 | 0.00 | 5.21 |
| 4 | α-D-Glucose 1-phosphate | 1.120 | 260.04 | 7.73 | 0.00 | 4.50 |
| 5 | Lipoxin C4 | 1.832 | 641.30 | 12.81 | 0.00 | 4.00 |
| 6 | 17-phenoxy trinor PGF2α ethyl amide | 1.875 | 431.27 | 5.43 | 0.00 | 3.45 |
| 7 | 5-HETE | 2.040 | 320.24 | 13.3 | 0.00 | 3.05 |
| 8 | Sphinganine-phosphate | 1.338 | 381.26 | 12.04 | 0.00 | 2.74 |
| 9 | α-D-Glucose | 1.045 | 180.06 | 0.78 | 0.00 | 2.63 |
| 10 | Deoxyuridine monophosphate (dUMP) | 1.536 | 308.04 | 0.97 | 0.01 | 2.41 |
| 11 | 4-aminohippurate | 1.117 | 222.10 | 3.76 | 0.00 | 1.84 |
| 12 | 1-Methyladenosine | 1.342 | 281.11 | 0.95 | 0.00 | 1.40 |
| 13 | Phenylglyoxylic acid | 1.242 | 150.03 | 0.55 | 0.00 | 1.02 |
| 14 | Geranylgeranyl PP | 1.407 | 450.20 | 12.35 | 0.00 | 0.98 |
| 15 | Androstenedione | 1.464 | 286.19 | 13.2 | 0.00 | 0.91 |
| 16 | Tetradecanedioic acid | 2.111 | 258.18 | 14.1 | 0.00 | 0.81 |
| 17 | Sphingosine 1-phosphate | 1.006 | 379.25 | 11.74 | 0.00 | 0.57 |
| 18 | PC(16:0) | 1.006 | 495.33 | 13.25 | 0.02 | -0.28 |
| 19 | PC(18:3) | 1.645 | 517.31 | 13.25 | 0.00 | -0.32 |
| 20 | PC(18:0) | 1.124 | 523.36 | 14.74 | 0.00 | -0.33 |
| 21 | Androsterone | 1.043 | 290.22 | 14.24 | 0.02 | -0.40 |
| 22 | Indoleacrylic acid | 1.142 | 187.06 | 3.8 | 0.00 | -0.40 |
| 23 | 12-amino-dodecanoic acid | 1.787 | 215.19 | 8.32 | 0.00 | -0.43 |
| 24 | psychosine sulfate | 1.461 | 541.30 | 12.76 | 0.00 | -0.43 |
| 25 | PC(20:3) | 1.627 | 545.35 | 14.74 | 0.00 | -0.44 |
| 26 | Uridine | 1.284 | 244.07 | 10.74 | 0.01 | -0.44 |
| 27 | 2-Hydroxyestrone | 1.213 | 286.15 | 10.21 | 0.00 | -0.46 |
| 28 | D-myo-Inositol-1,3,4,5-tetraphosphate | 1.302 | 499.92 | 0.57 | 0.01 | -0.57 |
| 29 | Deoxycorticosterone | 1.101 | 330.22 | 14.88 | 0 | -0.643 |
| 30 | N4-Acetylcytidine | 1.122 | 285.09 | 0.97 | 0.00 | -0.70 |
| 31 | Dihydroergotamine | 1.420 | 583.30 | 13.31 | 0.00 | -0.79 |
| 32 | 5-Methyluridine | 1.004 | 258.09 | 11.9 | 0.02 | -0.84 |
| 33 | deoxyguanosine 5'-monophosphate (dGMP) | 1.071 | 347.07 | 0.61 | 0.00 | -0.97 |
| 34 | ferulic acid | 1.342 | 194.06 | 8.65 | 0.00 | -1.14 |
| 35 | Chenodeoxycholic acid 3-sulfate | 1.945 | 472.26 | 11.73 | 0.00 | -1.18 |
| 36 | Androsterone sulfate | 1.035 | 370.18 | 7.98 | 0.01 | -1.26 |
| 37 | Abscisic Acid | 1.342 | 264.14 | 12.69 | 0.00 | -1.29 |
| 38 | Tryptophyl-Phenylalanine | 1.030 | 351.16 | 6.28 | 0.00 | -1.33 |
| 39 | Taurochenodeoxycholate-3-sulfate | 1.465 | 579.26 | 12.11 | 0.00 | -1.40 |
| 40 | L-Glutamine | 1.113 | 146.07 | 0.65 | 0.00 | -1.40 |
| 41 | 7-Oxo-11-dodecenoic acid | 1.346 | 212.14 | 8.75 | 0.01 | -1.52 |
| 42 | Bilirubin | 1.382 | 584.26 | 7.09 | 0.00 | -1.69 |
| 43 | PC(21:0) | 1.992 | 579.40 | 13.66 | 0.00 | -1.71 |
| 44 | Leukotriene C4 | 1.784 | 625.31 | 13.65 | 0.00 | -1.72 |
| 45 | C-6 NBD-dihydro-Ceramide | 1.671 | 577.38 | 12.73 | 0.00 | -1.98 |
| 46 | UDP-4-dehydro-6-deoxy-D-glucose | 1.322 | 548.05 | 0.95 | 0.00 | -2.05 |
| 47 | Galactitol 1-phosphate | 1.628 | 262.05 | 3.8 | 0.01 | -2.31 |
| 48 | L-Ascorbate 6-phosphate | 1.092 | 256.00 | 5.47 | 0.01 | -2.82 |

**Supplementary Table S6:** Differential metabolites in 200µM Ni^2+^-24h group.

| **No.** | **Name** | **VIP** | **mz** | **RT(min)** | **ttest** | **Log_2_FC** |
| --- | --- | --- | --- | --- | --- | --- |
| 1 | Uric acid | 2.033 | 168.03 | 0.8 | 0.02 | 4.34 |
| 2 | Deoxycholic acid | 2.381 | 392.29 | 12.4 | 0.00 | 3.45 |
| 3 | 3-Indoleacetic Acid | 1.861 | 175.07 | 3.271 | 0.00 | 3.26 |
| 4 | deoxyguanosine 5'-monophosphate (dGMP) | 1.788 | 347.07 | 0.628 | 0.01 | 2.93 |
| 5 | Cholic acid | 2.248 | 408.29 | 10.56 | 0.01 | 2.73 |
| 6 | 5'-Deoxy-5'-(methylthio)adenosine | 1.784 | 297.09 | 3.875 | 0.00 | 2.70 |
| 7 | 3-Indolebutyric acid | 2.113 | 203.10 | 6.157 | 0.00 | 2.05 |
| 8 | Dimethyl fumarate | 1.700 | 144.04 | 0.743 | 0.03 | 1.73 |
| 9 | Nicotinate D-ribonucleotide | 1.616 | 336.05 | 0.688 | 0.01 | 1.66 |
| 10 | PA(39:5) | 2.060 | 736.51 | 8.505 | 0.00 | 1.64 |
| 11 | Linoleic acid | 1.304 | 280.24 | 16.64 | 0.17 | 1.53 |
| 12 | γ-Linolenic Acid/α-Linolenic Acid | 2.081 | 278.22 | 15.79 | 0.02 | 1.50 |
| 13 | Dopamine | 1.945 | 153.08 | 1.183 | 0.00 | 1.17 |
| 14 | PC(22:6) | 2.369 | 567.33 | 12.647 | 0.00 | 1.10 |
| 15 | PC(20:4) | 1.815 | 543.35 | 12.694 | 0.006 | 0.882 |
| 16 | PI(44:0) | 1.792 | 978.72 | 9.585 | 0.01 | 0.86 |
| 17 | Indole-3-carboxylic acid | 1.605 | 161.05 | 1.373 | 0.02 | 0.82 |
| 18 | α-D-Glucose/D-Fructose | 2.071 | 180.06 | 0.65 | 0.02 | 0.70 |
| 19 | LysoPE(18:3) | 2.835 | 475.27 | 13.06 | 0 | 0.64 |
| 20 | Glycerophospho-N-Palmitoyl Ethanolamine | 2.854 | 453.29 | 12.788 | 0 | 0.638 |
| 21 | Nonanedioic acid | 1.446 | 188.10 | 6.9 | 0.13 | 0.60 |
| 22 | LysoPC(22:5) | 1.714 | 569.35 | 13.008 | 0.005 | 0.541 |
| 23 | Glucoheptonic acid | 2.658 | 226.07 | 0.65 | 0.00 | 0.53 |
| 24 | L-α-Hydroxyisovaleric acid | 1.240 | 118.06 | 3.33 | 0.20 | 0.49 |
| 25 | Glycerophospho-N-Oleoyl Ethanolamine | 1.325 | 479.30 | 13.222 | 0.047 | 0.409 |
| 26 | PC(18:2) | 2.145 | 519.34 | 12.639 | 0.00 | 0.40 |
| 27 | PC(15:0) | 2.439 | 481.32 | 14.27 | 0 | 0.368 |
| 28 | LysoPE(20:5) | 1.808 | 499.27 | 12.578 | 0.01 | 0.37 |
| 29 | LysoPE(18:2) | 1.428 | 477.29 | 12.582 | 0.03 | 0.33 |
| 30 | Glycerophospho-N-Arachidonoyl Ethanolamine | 1.624 | 501.29 | 12.425 | 0.02 | 0.31 |
| 31 | L-Leucine | 1.253 | 131.10 | 1.192 | 0.04 | 0.25 |
| 32 | LysoPC(18:3) | 2.941 | 517.32 | 13.129 | 0 | 0.228 |
| 33 | Palmitoyl-L-carnitine | 1.445 | 399.34 | 13.073 | 0.041 | 0.185 |
| 34 | PC(16:0) | 1.642 | 495.34 | 13.127 | 0.015 | 0.176 |
| 35 | Phenylglyoxylic acid | 1.606 | 150.03 | 0.556 | 0.01 | -0.10 |
| 36 | Lipoxin C4 | 1.447 | 641.30 | 14.68 | 0.13 | -0.25 |
| 37 | Sphingosine 1-phosphate | 2.134 | 379.25 | 11.67 | 0.01 | -0.28 |
| 38 | PC(17:1) | 1.658 | 507.33 | 13.6 | 0.07 | -0.28 |
| 39 | PC(17:0) | 1.854 | 509.35 | 14.67 | 0.04 | -0.31 |
| 40 | Linoleyl carnitine | 1.620 | 423.34 | 12.653 | 0.02 | -0.33 |
| 41 | Sphinganine | 2.106 | 301.30 | 11.293 | 0.00 | -0.42 |
| 42 | Hypoxanthine | 2.629 | 136.04 | 0.981 | 0.00 | -0.60 |
| 43 | Octanoylcarnitine | 1.766 | 287.21 | 7.886 | 0.01 | -0.64 |
| 44 | PC(18:3) | 1.656 | 517.33 | 13.55 | 0.07 | -0.64 |
| 45 | N-Oleoyl-L-Serine | 1.487 | 369.29 | 11.286 | 0.02 | -0.69 |
| 46 | Decanoyl-L-carnitine | 2.096 | 315.24 | 9.382 | 0.00 | -0.77 |
| 47 | 4-Nitroquinoline-1-oxide | 1.565 | 190.03 | 0.631 | 0.01 | -0.83 |
| 48 | Phytosphingosine | 1.975 | 317.29 | 10.103 | 0.01 | -0.84 |
| 49 | PC(19:0) | 2.041 | 551.36 | 13.99 | 0.02 | -0.87 |
| 50 | D-Glucose 6-sulfate | 1.824 | 260.02 | 4.19 | 0.04 | -0.90 |
| 51 | 15(S)-15-methyl PGF2α ethyl amide | 1.347 | 395.30 | 11.641 | 0.04 | -0.92 |
| 52 | N-palmitoyl histidine | 1.406 | 393.29 | 11.28 | 0.02 | -0.99 |
| 53 | PE(20:3) | 1.267 | 503.30 | 13.01 | 0.19 | -1.08 |
| 54 | N-docosanoyl taurine | 2.193 | 447.36 | 12.664 | 0.001 | -1.096 |
| 55 | N-stearoyl taurine | 1.419 | 391.27 | 10.674 | 0.03 | -1.12 |
| 56 | Sphinganine-phosphate | 1.633 | 381.26 | 11.96 | 0.08 | -1.13 |
| 57 | Glycocholic Acid | 1.662 | 465.32 | 15.05 | 0.07 | -1.16 |
| 58 | corticosterone | 1.626 | 346.21 | 15.88 | 0.08 | -1.20 |
| 59 | Hippuric acid | 1.397 | 179.06 | 4.732 | 0.05 | -1.29 |
| 60 | Leukotriene C4 | 1.378 | 625.31 | 13.35 | 0.15 | -1.32 |
| 61 | Pipecolic acid | 1.739 | 129.08 | 0.91 | 0.01 | -1.34 |
| 62 | Leukotriene C4 methyl ester | 1.481 | 639.30 | 14.57 | 0.12 | -1.36 |
| 63 | Indoxylsulfuric acid | 1.837 | 213.01 | 4.61 | 0.04 | -1.42 |
| 64 | 25-hydroxy-cholesterol | 2.205 | 405.35 | 10.202 | 0.00 | -1.55 |
| 65 | N-palmitoyl glutamic acid | 1.255 | 385.28 | 10.03 | 0.01 | -2.14 |
| 66 | Cinnamic acid | 2.124 | 148.05 | 2 | 0.01 | -2.95 |

**Supplementary Table S7:** Differential metabolites in 200µM Ni^2+^-48h group.

| **No.** | **Name** | **VIP** | **mz** | **RT(min)** | **ttest** | **Log_2_FC** |
| --- | --- | --- | --- | --- | --- | --- |
| 1 | Glycerophosphocholine | 2.136 | 257.10 | 0.753 | 0.00 | 5.35 |
| 2 | deoxyguanosine 5'-monophosphate (dGMP) | 1.654 | 347.07 | 0.628 | 0.00 | 4.47 |
| 3 | 5'-Deoxy-5'-(methylthio)adenosine | 1.786 | 297.09 | 3.875 | 0.00 | 3.50 |
| 4 | PC(18:3) | 2.039 | 517.32 | 11.929 | 0.00 | 3.11 |
| 5 | 3-Indoleacetic Acid | 1.157 | 175.07 | 3.271 | 0.03 | 3.00 |
| 6 | Pyroglutamic acid | 2.109 | 129.04 | 0.984 | 0.00 | 2.61 |
| 7 | LysoPE(18:3) | 1.948 | 475.27 | 11.866 | 0.00 | 2.29 |
| 8 | Arachidonoyl dopamine | 1.646 | 439.31 | 13.423 | 0.001 | 2.229 |
| 9 | Glycocholic Acid | 1.201 | 467.34 | 14.975 | 0.023 | 2.167 |
| 10 | Nicotinate D-ribonucleotide | 1.715 | 336.05 | 0.688 | 0.00 | 2.03 |
| 11 | 3-Methylxanthine/7-Methylxanthine/1-Methylxanthine | 1.921 | 166.05 | 0.69 | 0.02 | 1.90 |
| 12 | 3-Indolebutyric acid | 1.487 | 203.10 | 6.157 | 0.00 | 1.85 |
| 13 | PGF2α Alcohol methyl ether | 1.094 | 354.28 | 15.807 | 0.023 | 1.701 |
| 14 | Phytosphingosine | 3.104 | 317.29 | 10.103 | 0.00 | 1.63 |
| 15 | LysoPC(22:5) | 2.37 | 569.35 | 13.374 | 0 | 1.576 |
| 16 | PGF1α | 1.022 | 356.27 | 12.125 | 0.03 | 1.45 |
| 17 | PC(14:0) | 1.388 | 467.30 | 13.803 | 0.009 | 1.445 |
| 18 | LysoPE(20:5) | 1.115 | 499.27 | 12.346 | 0.04 | 1.41 |
| 19 | LysoPC(22:6) | 1.629 | 567.33 | 12.647 | 0.001 | 1.24 |
| 20 | Deoxycholic acid | 1.676 | 392.29 | 15.69 | 0.05 | 1.19 |
| 21 | Dopamine | 1.390 | 153.08 | 1.183 | 0.00 | 1.13 |
| 22 | PE(20:3) | 2.136 | 503.30 | 13.2 | 0.01 | 1.04 |
| 23 | LysoPE(22:5) | 2.019 | 527.30 | 13.05 | 0.01 | 1.01 |
| 24 | Indole-3-carboxylic acid | 1.459 | 161.05 | 1.373 | 0.01 | 0.88 |
| 25 | PE(16:0) | 2.415 | 453.29 | 13.063 | 0 | 0.824 |
| 26 | Taurocholic acid | 1.056 | 515.30 | 12.141 | 0.03 | 0.76 |
| 27 | EPA | 1.712 | 302.22 | 16.535 | 0.001 | 0.756 |
| 28 | PE(16:1) | 1.701 | 451.27 | 12.16 | 0.05 | 0.75 |
| 29 | PC(18:2) | 2.478 | 519.34 | 12.639 | 0 | 0.719 |
| 30 | Glycerophospho-N-Oleoyl Ethanolamine | 1.918 | 479.30 | 13.222 | 0 | 0.689 |
| 31 | Glycerophospho-N-Palmitoyl Ethanolamine/PC(13:0) | 2.538 | 453.29 | 13.12 | 0.00 | 0.69 |
| 32 | Adrenaline | 1.384 | 183.09 | 0.641 | 0.00 | 0.67 |
| 33 | Sphinganine | 2.446 | 301.30 | 11.293 | 0.00 | 0.67 |
| 34 | α-D-Glucose/D-Fructose | 1.805 | 180.06 | 0.65 | 0.03 | 0.65 |
| 35 | PC(15:0) | 2.344 | 481.32 | 14.31 | 0.00 | 0.54 |
| 36 | Cystathionine | 1.747 | 222.06 | 9.999 | 0.00 | 0.54 |
| 37 | PC(18:1) | 1.752 | 521.35 | 13.532 | 0 | 0.522 |
| 38 | LysoPE(20:4) | 1.687 | 501.29 | 12.425 | 0.00 | 0.48 |
| 39 | Glycerophospho-N-Palmitoyl Ethanolamine | 1.049 | 453.29 | 12.788 | 0.026 | 0.469 |
| 40 | Phenylpyruvic acid | 1.535 | 164.05 | 1.03 | 0.01 | 0.41 |
| 41 | LysoPE(22:6) | 1.465 | 525.29 | 12.603 | 0.00 | 0.37 |
| 42 | L-Leucine | 1.434 | 131.10 | 1.192 | 0.02 | 0.35 |
| 43 | Taurodeoxycholic acid | 1.174 | 499.27 | 12.578 | 0.02 | 0.32 |
| 44 | LysoPE(18:1) | 1.089 | 479.30 | 13.47 | 0.032 | 0.318 |
| 45 | Palmitoyl-L-carnitine | 1.281 | 399.34 | 13.073 | 0.009 | 0.307 |
| 46 | PC(16:0) | 1.477 | 495.34 | 13.127 | 0.003 | 0.298 |
| 47 | LysoPC(18:3) | 2.22 | 517.32 | 13.129 | 0 | 0.277 |
| 48 | L-Phenylalanine | 1.337 | 165.08 | 1.942 | 0.02 | 0.25 |
| 49 | α-Linolenoyl Ethanolamide | 1.571 | 321.26 | 13.343 | 0.006 | 0.174 |
| 50 | Phenylglyoxylic acid | 1.594 | 150.03 | 0.556 | 0.01 | -0.09 |
| 51 | Acetylcarnitine | 1.160 | 203.12 | 0.731 | 0.02 | -0.43 |
| 52 | Linoleyl carnitine | 1.721 | 423.34 | 12.653 | 0.001 | -0.466 |
| 53 | Hypoxanthine | 2.051 | 136.04 | 0.981 | 0.00 | -0.53 |
| 54 | Octanoylcarnitine | 1.457 | 287.21 | 7.886 | 0.02 | -0.56 |
| 55 | Decanoyl-L-carnitine | 1.639 | 315.24 | 9.382 | 0.01 | -0.65 |
| 56 | 4-Nitroquinoline-1-oxide | 1.533 | 190.03 | 0.631 | 0.00 | -0.90 |
| 57 | Arachidyl carnitine | 1.276 | 455.40 | 15.102 | 0.029 | -1.05 |
| 58 | 2-keto valeric acid | 2.043 | 116.05 | 1.9 | 0.01 | -1.51 |
| 59 | N-stearoyl taurine | 1.642 | 391.27 | 10.674 | 0.01 | -1.58 |
| 60 | Pipecolic acid | 1.536 | 129.08 | 0.91 | 0.00 | -1.67 |

**Supplementary Table S8:** The semiquantitative data of RRM2 protein in untreated and Ni^2+^-treated groups for four types of L929 cells.

| **Type of cells** | **Groups** | **GAPDH-1** | **GAPDH-2** | **GAPDH-3** | **RRM2-1** | **RRM2-2** | **RRM2-3** |
| --- | --- | --- | --- | --- | --- | --- | --- |
| *Rrm2-*silenced control cells | Untreated | 17558 | 19682 | 24174 | 8246 | 6710 | 11249 |
|  | 100µM Ni^2+^-12h | 18349 | 18261 | 15731 | 13778 | 15842 | 14562 |
|  | 200µM Ni^2+^-12h | 16508 | 17756 | 19847 | 19292 | 20174 | 19019 |
| *Rrm2-*silenced cells | Untreated | 12430 | 16943 | 17590 | 3095 | 4601 | 2512 |
|  | 100µM Ni^2+^-12h | 13507 | 21977 | 18363 | 5837 | 6694 | 8045 |
|  | 200µM Ni^2+^-12h | 15254 | 16998 | 16675 | 6672 | 9294 | 8019 |
| *Rrm2*-overexpression control cells | Untreated | 14125 | 17532 | 16474 | 3323 | 3443 | 3662 |
|  | 100µM Ni^2+^-12h | 16485 | 19586 | 18496 | 5169 | 7011 | 6421 |
|  | 200µM Ni^2+^-12h | 20977 | 19316 | 21735 | 12830 | 12169 | 14134 |
| *Rrm2*-overexpression cells | Untreated | 27331 | 19083 | 19911 | 11590 | 10399 | 9129 |
|  | 100µM Ni^2+^-12h | 15398 | 15460 | 18387 | 10486 | 10747 | 13545 |
|  | 200µM Ni^2+^-12h | 23307 | 20102 | 18538 | 19175 | 18051 | 15285 |

**Supplementary Fig. S1:** The biosynthesis of unsaturated fatty acids pathway [26] in which gene/protein pair and metabolites exhibited upstream-downstream relationships in the interaction between Ni^2+^ and L929 cells.

**Supplementary Fig. S2:** The cysteine and methionine metabolism pathway [26] in which gene/protein pair and metabolites exhibited upstream-downstream relationships in the interaction between Ni^2+^ and L929 cells.

**Supplementary Fig. S3:** The amino sugar and nucleotide sugar metabolism pathway [26] in which gene/protein pair and metabolites exhibited upstream-downstream relationships in the interaction between Ni^2+^ and L929 cells.

**Supplementary Fig. S4:** The porphyrin metabolism pathway [26] in which gene/protein pair and metabolites exhibited upstream-downstream relationships in the interaction between Ni^2+^ and L929 cells.

**Supplementary Fig. S5:** The glycerophospholipid metabolism pathway [26] in which gene/protein pair and metabolites exhibited upstream-downstream relationships in the interaction between Ni^2+^ and L929 cells.

**Supplementary Fig. S6:** Cell cycle analysis results in untreated and Ni^2+^-treated groups for four types of L929 cells. (**A**) *Rrm2*-silenced control L929 cells, untreated group. (**B**) *Rrm2-*silenced control L929 cells, 100μM Ni^2+^-treated group. (**C**) *Rrm2*-silenced control L929 cells, 200μM Ni^2+^-treated group. (**D**) *Rrm2*-silenced L929 cells, untreated group. (**E**) *Rrm2-*silenced L929 cells, 100μM Ni^2+^-treated group. (**F**) *Rrm2*-silenced L929 cells, 200μM Ni^2+^-treated group. (**G**) *Rrm2-*overexpressing control L929 cells, untreated group. (**H**) *Rrm2-*overexpressing control L929 cells, 100μM Ni^2+^-treated group. (**I**) *Rrm2-*overexpressing control L929 cells, 200μM Ni^2+^-treated group. (**J**) *Rrm2-*overexpressing L929 cells, untreated group. (**K**) *Rrm2-*overexpressing L929 cells, 100μM Ni^2+^-treated group. (**L**) *Rrm2-*overexpressing L929 cells, 200μM Ni^2+^-treated group.
